# Development of an automated optimal distance feature-based decision system for diagnosing knee osteoarthritis using segmented X-ray images

**DOI:** 10.1016/j.heliyon.2023.e21703

**Published:** 2023-11-03

**Authors:** Kaniz Fatema, Md Awlad Hossen Rony, Sami Azam, Md Saddam Hossain Mukta, Asif Karim, Md Zahid Hasan, Mirjam Jonkman

**Affiliations:** a Health Informatics Research Lab, Department of Computer Science and Engineering, Daffodil International University, Dhaka, 1341, Bangladesh; b Faculty of Science and Technology, Charles Darwin University, Darwin, NT, 0909, Australia; c Department of Computer Science and Engineering, United International University, Dhaka, 1212, Bangladesh

**Keywords:** Knee osteoarthritis, Segmentation, Feature extraction, Machine learning, Feature selection, Proposed features, Decision making system

## Abstract

Knee Osteoarthritis (KOA) is a leading cause of disability and physical inactivity. It is a degenerative joint disease that affects the cartilage, cushions the bones, and protects them from rubbing against each other during motion. If not treated early, it may lead to knee replacement. In this regard, early diagnosis of KOA is necessary for better treatment. Nevertheless, manual KOA detection is time-consuming and error-prone for large data hubs. In contrast, an automated detection system aids the specialist in diagnosing KOA grades accurately and quickly. So, the main objective of this study is to create an automated decision system that can analyze KOA and classify the severity grades, utilizing the extracted features from segmented X-ray images. In this study, two different datasets were collected from the Mendeley and Kaggle database and combined to generate a large data hub containing five classes: Grade 0 (Healthy), Grade 1 (Doubtful), Grade 2 (Minimal), Grade 3 (Moderate), and Grade 4 (Severe). Several image processing techniques were employed to segment the region of interest (ROI). These included Gradient-weighted Class Activation Mapping (Grad-Cam) to detect the ROI, cropping the ROI portion, applying histogram equalization (HE) to improve contrast, brightness, and image quality, and noise reduction (using Otsu thresholding, inverting the image, and morphological closing). Besides, the focus filtering method was utilized to eliminate unwanted images. Then, six feature sets (morphological, GLCM, statistical, texture, LBP, and proposed features) were generated from segmented ROIs. After evaluating the statistical significance of the features and selection methods, the optimal feature set (prominent six distance features) was selected, and five machine learning (ML) models were employed. Additionally, a decision-making strategy based on the six optimal features is proposed. The XGB model outperformed other models with a 99.46 % accuracy, using six distance features, and the proposed decision-making strategy was validated by testing 30 images.

## 1 Introduction

Knee osteoarthritis (KOA) is a degenerative joint disease and a global source of disability [1]. It mainly affects the cartilage, the protective tissue covering the ends of the bones. Normal or healthy cartilage enables movement of the bones, which form a joint while keeping them from rubbing against each other [2]. In KOA, the top layer of cartilage is broken down, causing the bones to rub together, which can be exceedingly painful [3]. Osteo Arthritis frequently affects the knee, spine, hip, and foot joints. It can be primary or secondary. Primary KOA occurs in older people and may be hereditary or due to aging. Secondary KOA is related to trauma, diabetes, high-impact sports, and rheumatoid arthritis and tends to happen earlier in life. The World Health Organization (WHO) estimates that 9.6 % of men and 18.0 % of women over 60 have osteoarthritis. 80 % of these have mobility challenges, and 25 % have trouble doing their daily tasks. Another study estimates that the number of people over 45 years old with KOA is expected to rise from 13.8 % to 15.7 % by 2032 [4]. Severe pain, restricted joint movement, and notably stiff joints in the morning are the main symptoms of KOA which can interfere with the patient's ability to perform routine activities [5]. Pain causes depression, while chronic pain is physically and emotionally stressful. Chronic stress has been shown to alter the amounts of brain and nervous system chemicals. Besides, pain is exacerbated by depression, which impairs one's capacity to deal with and cope with the discomfort. Moreover, it also causes hypertension and badly affects mental health. Several researchers focused [[6], [7], [8]] on preventing hyper-tension and mental health issues in their work. However, KOA is diagnosed based on pain and a combination of clinical and radiographic symptoms. Though pain is the key symptom, it is subjective and difficult to quantify [9]. Several techniques exist to classify osteoarthritis stages, but radiographic findings can correspond to several symptoms [10]. There may be an unexplained discrepancy between radiographic findings and clinical presentation [11]. Magnetic resonance imaging (MRI) can detect knee morphology better than radiography. It provides a detailed image of the structure and composition of the knee, but it is more expensive than radiography [12]. Though magnetic resonance imaging (MRI) has shown potential [13], most orthopedic surgeons rely on standard weight-bearing radiography. Moreover, X-rays help to detect degeneration of articular cartilage, narrowing of articular space between adjacent bones, and development of bone spurs [14] and are commonly available, affordable, and safe [15]. The Kellgren & Lawrance (KL) OA classification is a popular classification technique [16,17]. Radiologists can estimate the severity of the disease by measuring joint space narrowing, osteoporotic formations, and subchondral sclerosis, grading the severity from 0 to 4. In particular, the joint knee space width (JSW) has proved crucial in determining the development and severity of OA [[18], [19], [20]]. This knee JSW helps to predict the progression of OA. Several researchers employed different deep learning and machine learning methods to the limited x-ray image datasets for classifying Grades 0 to 4 [5,[21], [22], [23], [24], [25], [26], [27], [28]]. Nevertheless, their model did not obtain satisfactory accuracy in multi-class classification. So, accurately classifying OA stages using X-ray images is still difficult, time-consuming, and error prone. Moreover, there does not exist any study where researchers introduce any optimal segmentation method and propose an automated decision system using only the extracted medical features value without facing any complexity issue. From this motivation, we considered a need for computer-assisted diagnostic tools for medical professionals to diagnose knee OA severity consistently and automatically. Increasing the validity and reproducibility of X-ray interpretation for OA diagnosis would be highly beneficial. This research aimed to develop a system that automates the classification of the KOA using optimal extracted features from segmented knee images and compare its accuracy to earlier research. In this research, we obtained a total of 8,660 images after combining two different knee datasets with five classes, including (Grade 0), doubtful (Grade 1), minimal KOA (Grade 2), moderate KOA (Grade 3), and severe KOA (Grade 4). The dataset is preprocessed using several preprocessing techniques. After enhancing images, 711 image outliers were found in the combined image dataset, which was removed. Six images from each class, 30 images in total, were kept separately for testing purposes. Consequently, 7,919 images remained. These were segmented and split according to an 80:20 ratio for training and testing. Knee joint segmentation and preprocessing methods are carried out to ensure optimal visibility of the knee joint. Accurate knee joint segmentation for all images is challenging due to different intensity levels. The segmented images were split into Medial side and Lateral side, resulting in 15,838 images from which features were extracted. Six sets of different handcrafted features are created: morphological features (16), GLCM features (12), statistical features (4), texture features (4), LBP features (4), and proposed features (12). Five ML models are employed to identify the best feature set. After initial experimentation, it was found that the XGBoost (XGB) model outperformed the other models for the proposed feature set. Then several feature selection techniques are applied to the proposed features, and the top six features are identified. We propose a decision system based on these six features to diagnose the KOA grade and use 30 unseen X-ray images to test it. This work is done based on image segmentation, image processing, extracting handcrafted medical features, and knowledge discovery following the values of the extracting features. However, in this section, a theoretical framework is also shown in Table 1, Table 2 that presents the working process of conducting the literature review. In general, conducting a systematic review is an appropriate method for collecting existing studies and identifying gaps that may propose a new area of research. A systematic review was undertaken to compile a summary of extant KOA-based solutions that address the current obstacles to KOA classification. The review protocol is completed by conducting an exhaustive paper search. The search phase entails defining academic databases and search queries for locating eligible datasets and studies. Table 1, Table 2 depict the databases and queries utilized in the investigations.

**Table 1:** Searching for the electronic database.

Electronic Data hub	Type	URL
Google Scholar	Search Engine	https://scholar.google.com.au/
ResearchGate	Social networking site	https://www.researchgate.net/
IEEE Xplore	Digital library	https://ieeexplore.ieee.org/Xplore/home.jsp
Science Direct-Elsevier	Digital library	https://www.sciencedirect.com/
Springer	Digital library	https://www.springer.com/gp
Wiley online library	Digital library	https://www.wiley.com/en-au
MDPI	Digital library	https://www.mdpi.com/

**Table 2:** Search queries used for the systematic review.

Query no	Search queries
Q1	Knee Osteoarthritis (KOA) detection using X-ray images OR KOA grades multi-class classification
Q2	KOA identification using an improved CNN OR deep CNN model OR machine learning model
Q3	Automated KOA segmentation and grades identification method
Q4	Knee cartilage region detection using X-ray images
Q5	KOA ROIs segmentation from X-ray images
Q6	Handcrafted feature extraction and analysis from KOA X-ray images

## 2 Previous work

Since various diseases can affect human health, it generates several risk factors in the human body. In the majority of cases, elderly people face health problems. Besides, the health problems of adolescents are also a crucial health issue worldwide [29]. They can be considered a burden to the nation. At the same time, several researchers focused on analyzing the patients and disease conditions by employing the advantages of digital innovation, including social media and the internet [30,31]. Similarly, KOA-affected patients might be burdened and unbearable due to the risk factors of the disease. Hence, KOA diagnosis becomes a crucial task for the affected patients. However, in this section, we discuss studies that use X-ray imaging to categorize KOA, employing different machine learning and deep learning techniques. Teo et al. [21] extracted features from X-ray images using three transfer learning models: InceptionV3, Xception, and DenseNet201. They used about 1,000 X-ray images from the Osteoarthritis Initiative (OAI) dataset in five classes: Grades 0 to 4. When the DenseNet201's feature set was fed into the SVM ML model, it obtained 71.33 % accuracy in multiclass classification. Shivanand et al. [5] suggested a semi-automated approach to identify structural anomalies in the knee. The researchers collected around 200 knee X-ray images from several hospitals and utilized an active contour algorithm to segment the region of the knee X-ray image relevant for diagnosis. Several feature extraction methods were used, including statistical, texture, first four moments and shapes, and a random forest (RF) classifier was employed. The model distinguished normal and OA images with an accuracy of 87.92 %. However accurately determining the optimal features requires, a large data hub and an optimal classifier. Sikkandar et al. [22] introduced an automatic identification method for KOA images, using a deep Siames CNN technique and an unsupervised segmentation approach for the local center of mass (LCM). They utilized the GLCM matrix and first order statistics to extract the most important features from the segmented images. They used digital clinical X-ray images of 350 patients with all four grades of KOA. Their study showed that the neural network worked well on the LCM segmented images, with a validation accuracy of 93.2 %, and a multiclass classification accuracy of 72.01 %. The lack of a large data set and proper image processing is a limitation of their method. Subramonium et al. [23] introduced a classification algorithm based on local binary patterns (LBP) to identify OA in knee X-rays. They utilized 50 knee X-ray images. To classify knee X-ray images, histograms of LBP were computed using K-nearest neighbours (KNN) classifiers. The classification accuracies for normal and abnormal knee images and a mild or severe cases, were 95.24 % and 97.37 % respectively. However, there were several ways in which the accuracy could have been improved, such as applying proper image processing, and using a larger data hub. Rabbia et al. [24] introduced a deep learning-based feature extraction and classification technique to grade KOA. They used a total of 2000 images from the Mendeley IV dataset. After preprocessing and extraction of the ROI from the images, the proposed model feature, joint space width, was extracted from the ROI using LBP and CNN with the histogram of oriented gradient (HOG). They employed three ML models for multiclass classification: SVM, RF, and KNN. The HOG features descriptor provided approximately 97 % accuracy for classifying all four grades of KOA. Sulaimanet al [25] suggested an approach based on customized CenterNet with a pixel-wise voting scheme to extract the features automatically. They utilized DenseNet-201 as a base network for feature extraction. They employed two separate datasets of five grades (0–4): the OAI dataset for cross-validation and the Mendeley VI (2,000 images) dataset for training (1500 images) and testing (500 images). Their method achieved 99.14 % accuracy when tested with 500 images and 98.97 % accuracy for cross-validation. Although their model produced the best results, adequate image processing and experiments with merged datasets could possibly further improve the model's performance. Yunus et al. [26] proposed another radiographic image-based KOA image classification and localization method. They used the Mendeley “knee osteoarthritis severity grading dataset”, which contains 3,795 knee images of five grades. They transformed the images into three channels of LAB and extracted features using Darknet-53 and Alex Net. PCA was used to select the best features. The YOLOv2-ONNX model with chosen hyperparameters was used to classify the knee images. Their method resulted in a mean average precision (mAP) of 98 % in classification. Since there is a chance of enhancing performance, proper ROI segmentation is required. In another study [27], the same 1,650 images from the same dataset were used and two different models were applied. The first model was used for classifying normal (Grade 0–1) and KOA (Grade II-IV). The second model categorized the severity as normal (Grade 0–1), non-severe (Grade II), and severe (Grade III-IV). They applied several augmentation techniques to balance the dataset and the YOLOv3 detection algorithm to diagnose classes. Their first model acquired 85 % accuracy, and the second model achieved 86.7 % accuracy in classifying the severity of KOA. Tiulpin et al. [28] used two datasets, the MOST (3,026 subjects) and the OAI (4,796 subjects) with five grades (Grade 0–4). The MOST dataset was used to train their models and the OAI dataset to test their models. They proposed a Siamese deep neural network for medical images with symmetry, making it more robust by reducing the number of learnable parameters. They patched the knee images on both the medial and lateral sides, resulting in a classification accuracy of 66.7 %. In this case, the model accuracy was very low compared to others, so it can be concluded that working with optimal features and ML models is probably a better approach. After reviewing the literature, it can be concluded that in most cases, the researchers used a single dataset, and their accuracy was in the range of 71.33 %–97.37 % for multiclass classification. The core limitation in most cases was the absence of a suitable ROI segmentation technique, feature selection, and evaluation with combined datasets. Several studies show that obtaining satisfactory results in KOA classification using a deep learning model is quite challenging. In this study these limitations are addressed by utilizing a combined dataset, identification of the optimal features, to construct a decision-making system for KOA multiclass classification. The limitations of the studies described above are summarized in Table 12.

## 3 Research aims and novel contributions

The proposed automated system follows a similar procedure as a radiologist in screening X-ray images. In a real knee X-ray screening, a radiologist mainly focuses on the knee joint gap, extracts different features, and provides high-accuracy classification results. If the X-ray images are taken using different sources, the ROI extraction process may change slightly due to the presence of different structures or intensity levels. It may be expected that the model will perform best in large-scale screening trials. An automated method performs better with a larger dataset. The main aim of this study is to determine the optimal features and develop a decision-making system based on these optimal features to classify KOA grades. A list of the research innovations and novel contributions is provided below:

1. Since different X-ray image datasets are collected using various sources and protocols, image qualities and details may be different. We combine two datasets with different properties and sizes of X-ray images to create a large data hub. This creates a challenge. The difficulty of dealing with various image dimensions is addressed in this research.
2. Several image processing techniques are used and the region of Interest (ROI) of the knee joint is extracted. These image processing techniques include Grade-CAM to find the ROI, histogram equalization to enhance the image quality, Otsu thresholding to convert the grayscale image into a binary image, Bitwise_NOT to invert the color of the pixels and morphological closing to denoise the images.
3. After dividing the segmented images into two groups: medial (M)and lateral (L), six different feature sets are introduced: morphological features (set1), GLCM features (set 2), statistical features (set 3), texture features (set 4), LBP features (set 5), and a proposed feature (set 6).
4. We proposed a set of 12 features, including distance features (M1, M2, M3, L1, L2, and L3), area features (medial and lateral -side area), peak values (medial and lateral peak value), and gradient features (medial and lateral gradient).
5. The six optimal features (M1, M2, M3, L1, L2, and L3) from the twelve previously proposed features are determined. The standard distance range and optimal median values of the range corresponding to the KOA grades are also identified.
6. An automated decision-making system is developed based on the six distance features (M1, M2, M3, L1, L2, and L3) and tested with 30 isolated segmented images to ensure its ability to determine the KOA grade correctly.

## 4 Medical analysis of knee X-ray image

Clinicians assess joint OA abnormalities by identifying knee joint space narrowing (JSN), sclerosis, cysts, attrition, chondrocalcinosis, and osteophytes in the knee X-ray images. The lateral femur compartment, the medial femur compartment, the lateral tibia compartment, and the medial tibia compartment are the locations where other OA characteristics are identified [32]. For KOA grading, clinicians measure JSN and osteophytes. The Kellgren-Lawrence method is the KOA grading approach that is most often utilized in clinical practice [33]. This technique categorizes KOA into five groups: healthy (Grade 0), doubtful (Grade 1), minimal (Grade 2), moderate (Grade 3), and severe (Grade 4). It is a semi-quantitative five-scale assessment of the overall severity of OA for the whole joint, taking into account several factors, such as osteophytes and JSN. Osteophytes are characterized by the expansion of bone and cartilage close to the joint [34]. The following radiological characteristics are considered evidence of KOA [35]:

1. The growth of osteophytes around the joint or on the tibial spine of the knee
2. Narrow joint cartilage and hardening of the bone under the cartilage.
3. Small areas of pseudocysts along with sclerosis walls are often found in the subchondral bone.
4. A change in the shape of the end of the bone, especially in the femur.

**Fig. 1:**
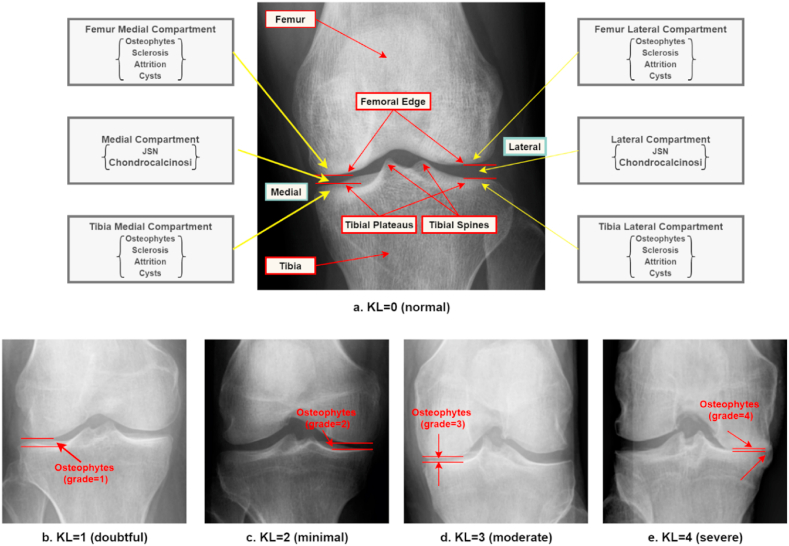
The example of the Kellgren–Lawrence (KL) scale: (a). Normal (grade 0), (b). Doubtful (grade 1), (c) Minimal (grade 2), (d) Moderate (grade 3), and (e) Severe (grade 4).

Fig. 1 shows examples of the different KOA grades. Table 3 describes the characteristics of each grade [33,36].

**Table 3:** Radiographic characteristics of KOA and the symptoms of the patients.

Grade	Characteristics of KOA (Grade0-Grade4)	Symptoms of patients
**Normal (Grade 0)**	There are no radiographic abnormalities associated with KOA. There is no JSN or active change. Fig. 1(a) shows that the joint space between two bones is normal and equal on both sides.	Patients are able to move without any discomfort or difficulty.
**Doubtful (Grade1)**	There is doubtful JSN, and osteoporotic lipping could happen. Fig. 1(b) shows that there is a possible JSN, and the presence of osteophytes.	During this stage, most patients don't feel any pain or discomfort.
**Minimal (Grade 2)**	There are definite osteophytes and JSN may be found. This is a mild case of KOA, where the cartilage is probably still in reasonable shape. Fig. 1(c) depicts the probability of JSN and the presence of osteophytes.	In this stage, people will feel pain and stiffness in the knee joint after a long day of walking.
**Moderate (Grade3)**	At this stage, definite JSN, some sclerosis, and numerous osteophytes are evident. Fig. 1(d) depicts JSN with many osteophytes.	The patient frequently has pain when moving. Moreover, joint stiffness is prevalent after lengthy periods of sitting and in the morning.
**Severe (Grade 4)**	This is considered the most severe stage of KOA. This stage has large osteophytes, significant sclerosis, and bone abnormalities. Moreover, synovial fluid, cartilage, and knee joint space are diminished. Fig. 1 (e) shows that the joint space between the bones is completely eliminated, the cartilage is nearly gone, and massive osteophytes are present.	The patient usually feels a lot of pain and discomfort when walking or moving the joint.

## 5 Methodology

**Fig. 2:**
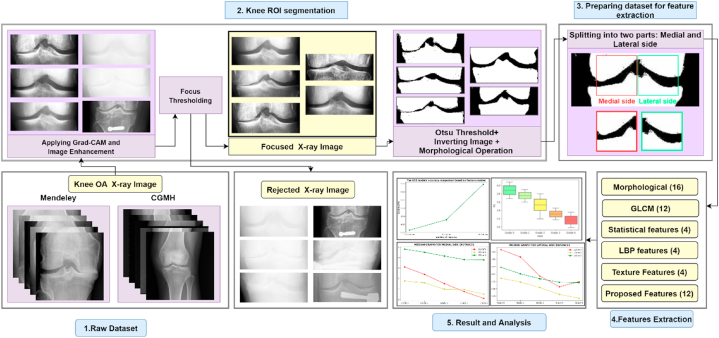
Methodology workflow.

Since mental health is directly related to physical health, addressing health issues such as chronic pain is crucial. Researchers prioritize innovation and the use of social media in various contexts to protect and improve people's mental health and quality of life [[37], [38], [39]]. However, we also focused on early KOA-grade detection to maintain people's physical and psychological health. Here, Fig. 2 illustrates the workflow of this research. It shows the different stages: combining two datasets with different quality images, image segmentation, preparing the dataset for feature extraction, feature extraction, and classification of KOA. Since we introduced and proposed several methods, all the methods consist of different parameters. In general, fine-tuning parameters are essential to obtain the optimal parameters for the greatest outcomes. As a result, we used the hyper-parameters technique to determine the optimal parameters that would perform best in our work. However, the details of each stage are described below.

### 5.1 Dataset preparation

We used two public data sets: CGMH Knee Osteoarthritis Images from Kaggle [40] and the Knee Osteoarthritis Severity Grading from Mendeley [41]. Each of these datasets included images of the five KOA grades: normal (Grade 0), doubtful (Grade 1), minimal KOA (Grade 2), moderate KOA (Grade 3), and severe KOA (Grade 4). The CGMH Knee Osteoarthritis dataset is well-balanced, with 80 knee images for each of the five classes. In contrast, the knee Osteoarthritis Severity dataset was unbalanced, with different numbers of knee X-ray images for different classes. After merging the two datasets, we had a total of 8,660 images, including 3,333 images for normal (Grade 0), 1,575 images for doubtful (Grade 1), 2,255 images for minimal (Grade 2), 1,166 images for moderate (Grade 3), and 331 images for severe (Grade 4). Table 4 gives an overview of the two datasets.

**Table 4:** Dataset description.

Class name	Mendeley dataset	Kaggle dataset	After integrating two datasets
Healthy	3,253	80	3,333
Doubtful	1,495	80	1,575
Minimal	2,175	80	2,255
Moderate	1,086	80	1,166
Severe	251	80	331
Total	8,260	400	8,660

**Fig. 3:**
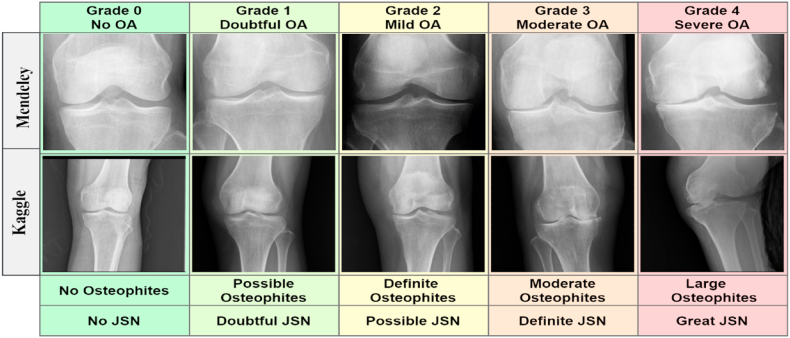
X-ray images of the five grades from two datasets.

**Fig. 4:**
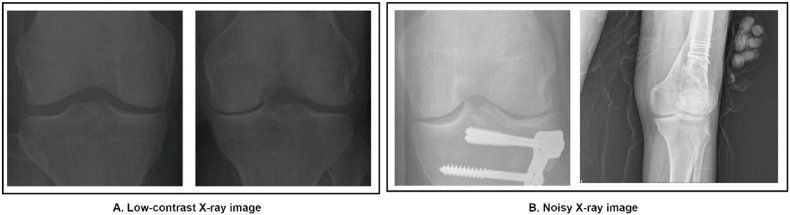
Challenges for the Knee X-ray images in the dataset: A. Low-contrast X-ray image and B. Noisy X-ray image.

Fig. 3 gives examples of dataset images, while Fig. 4 highlights some of the issues associated with some X-ray images.

Some of the challenges in interpreting knee X-rays are shown in Fig. 4 (A, B). Since these x-ray images are taken with different devices, the datasets contain a variety of low contrast and noisy images. Improving image quality and eliminating noise from the images is important for accurate diagnosis.

### 5.2 ROI segmentation

**Fig. 5:**
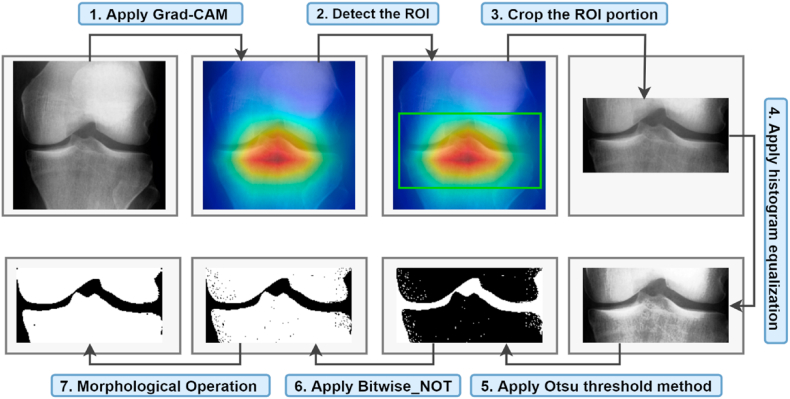
ROI segmentation process.

Detecting and extracting the knee joint space from the X-ray images should be the first step in identifying anomalies. Generally, segmentation is regarded as crucial for the automated interpretation of medical images [42]. Enhancing the X-ray images can result in a more precise ROI segmentation. Several image processing techniques can be used to improve segmentation of the ROI of X-ray images. Additionally, irrelevant portions of the X-ray images should be removed before ROI segmentation, as these may hinder the ROI extraction process. This section describes determining the ROI using GradeCAM, image enhancement, and noise reduction to improve image quality. So, we followed hyper-parameters tunning methods to obtain the optimal outputs. Fig. 5 illustrates the knee image processing techniques used in this study.

#### 5.2.1 Grad-CAM

**Fig. 6:**
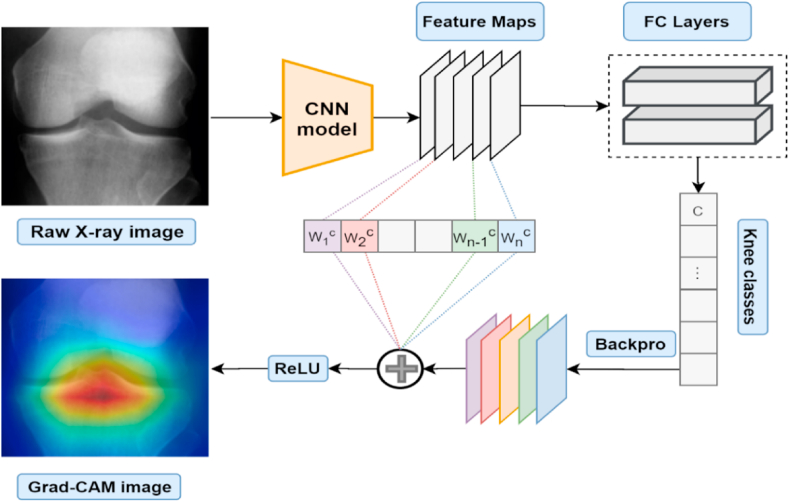
Grad-CAM overview.

The Grad-CAM technique is utilized to extract a feature map for deep neural networks and the attention mechanism is then employed to extract high-level attention maps. As a visual representation of a deep neural network, the attention map draws attention to the part of the image that is crucial for determining the target class. Grad-CAM is an alternative to the global average pooling (GAP) layer that relies on gradient computations [43]. Fig. 6 depicts Grade-CAM in action. Grade-CAM is a technique for merging feature maps, utilizing gradient weights without modifying the network topology. It permits any gradient to flow into the final convolutional layer to create an explanation map that highlights the sections of the image that are important for class prediction [44]. Also, it is a well-known method that is mostly used to detect ROIs when diagnosing KOA [45,46]. So, the overall calculation process demonstrated below [43].

The Grad-CAM technique computes the gradient of the class score $x^{n}$ with respect to the feature map of the last convolution layer while n denotes the class and $E_{pq}^{r}$ represents activation of the cell at spatial location p: (equation no 1)

(1) $$\frac{\partial x^{n}}{\partial E_{pq}^{r}}$$

It uses global-average-pooling gradients to get weights $W_{r}^{n}$, when M is the total number of feature map cells (equation no 2)

(2) $$W_{r}^{n}=\frac{1}{M}\sum_{p}\sum_{q}\frac{\partial x^{n}}{\partial E_{pq}^{r}}$$

Grad-CAM generalizes visual explanations using a weighted combination of feature maps with ReLU (equation (3)).

(3) $$L_{Grad-CAM}^{n}=ReLU\left(\sum_{r}W_{r}^{n}E^{r}\right)$$

in Equation (4), the weight $\alpha_{r}^{n}$ represents a partial linearization of the deep network downstream from E,M is the total number of feature map cells, $x^{n}$ is an activation class score for class n, and $E_{pq}^{r}$ represents activation of the cell at spatial location p.

(4) $$\alpha_{r}^{n}=\frac{1}{M}\sum_{p}\sum_{q}\frac{\partial x^{n}}{\partial E_{pq}^{r}}$$

**Fig. 7:**
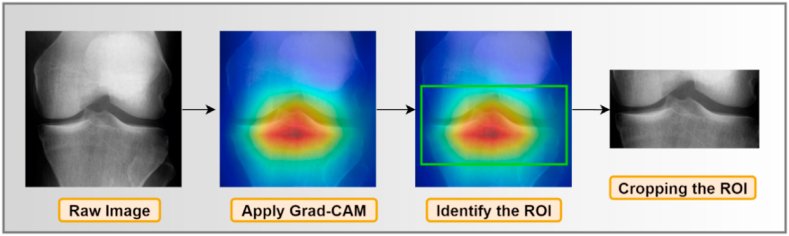
Output of the Grad-CAM process.

Grad-CAM assigns priority values to each neuron for a specific choice using the gradient information flowing into the last convolutional layer of the CNN model. Fig. 7 shows the output of the Grad-CAM process.

After acquiring the ROI through Grad-CAM, we manually crop the images to dimensions of 128 × 128 pixels, eliminating irrelevant regions from the images.

#### 5.2.2 Image enhancement

The HE technique is applied to the cropped image to enhance the image quality. The HE technique summarizes the intensities in an image and spreads out the gray levels of an image so that they are evenly distributed over their range. It modifies the brightness and contrast of an image [47]. By remapping the brightness values, this enhancement improves image contrast, equalizing the intensity values to the whole histogram range [48]. Each pixel of the input image is translated to a pixel of the output image [49]. The procedure may be described as follows (from equations no 5 to 9):

(5) $$P_{r}=\frac{Number\;of\;pixels\;with\;intensity\;\left(r_{q}\right)}{total\;number\;of\;pixels\;\left(r\right)}$$

where r=0,1….,N−1 are the gray level ranges' values. The probability of the density function, $pdf\left(Y_{q}\right)\text{,}$ for an image Y(a,b), is given by:

(6) $$Pdf\left(Y_{q}\right)=\frac{r^{q}}{r}$$

where, $r^{q}$ is the number of pixels with intensity level $Y_{q}$ in the input image Y and r denotes the total number of pixels. The proportion of pixels with a particular intensity $Y_{q}$ is represented by $pdf\left(Y_{q}\right)$ which is related to the image's histogram. The current cumulative density function e(x) is given by.

(7) $$e\left(x\right)=\sum_{i=0}^{q}p\left(Y_{i}\right)$$

The intensity values are remapped over the dynamic range, $\left(Y_{0},Y_{N-1}\right)$, by utilizing e(x) to transform the data, f(x), for example, a transformation function which depends on e(x) is given by

(8) $$f\;\left(x\right)=Y_{0}+\left(Y_{N-1}-Y_{0}\right)*e\left(x\right)$$

The output image, V={V(a,b)}, is then given by:

(9) V=f(x)={f(Y(a,b)∀Y(a,b)∈Y}

**Fig. 8:**
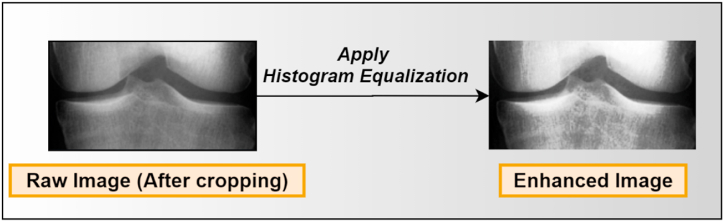
Output of the HE techniques.

The HE approach uses the picture's pixels to create a higher-quality image by changing their value. This technique helps to generate an enhanced X-ray image dataset for further processing. The output of the HE process is shown in Fig. 8.

#### 5.2.3 Focus filtering

Though the HE technique helps to improve image quality, there may still be blurring and unwanted noise. Therefore, the Laplace variance threshold approach is applied to the merged dataset to differentiate the X-ray images based on their blurriness and texture clarity [50,51]. In this study [52], researchers utilized this method to eliminate unwanted knee X-ray images from their generated dataset. The Laplacian of the image, which is the image's second derivative, is commonly utilized for edge detection. The following kernel (Equation. 10) is used to approximate the Laplacian for a random grayscale image I of size p×q:

(10) $$T=\left(\begin{array}{ccc} 0 & -1 & 0\\ -1 & 4 & -1\\ 0 & -1 & 0 \end{array}\right)$$

If U(I) is the convolution of image I with the Laplacian kernel T with a size of p×q,where $U{\left(I\right)}_{\alpha }$ represents an average of the values for $U{\left(I\right)}_{x,y}$ (Equation (11)). So, in the final step the focus metric is calculated as the difference between the absolute values of the convolved image (Equation (12)):

(11) $$U{\left(I\right)}_{\alpha }=\frac{1}{p\times q}\sum_{x=1}^{p}\sum_{y=1}^{q}\left|U{\left(I\right)}_{x,y}\right|$$

(12) $$U{\left(I\right)}_{var}=\sum_{x=1}^{p}\sum_{y=1}^{q}{\left[\left|U{\left(I\right)}_{x,y}\right|-U{\left(I\right)}_{\alpha }\right]}^{2}$$

**Fig. 9:**
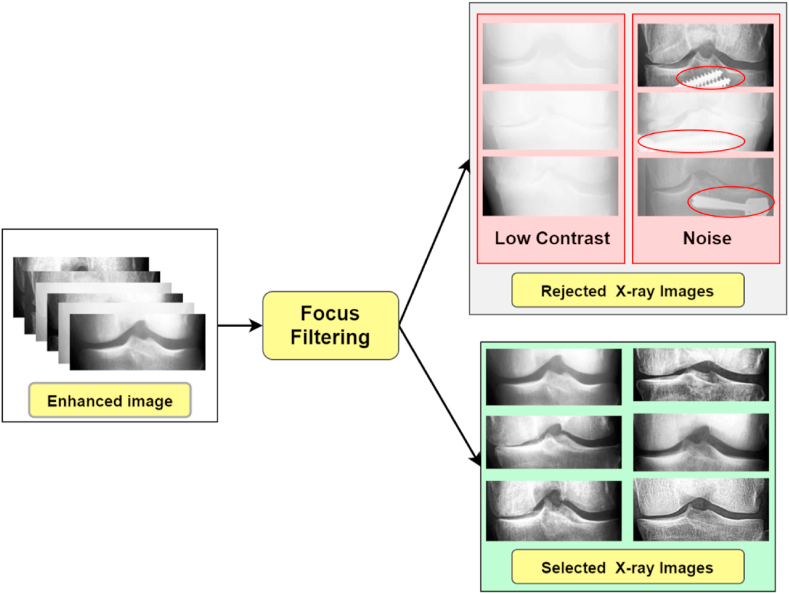
Rejected outliers from enhanced data hub, applying focus filtering.

After testing with multiple parameters, we use a variance threshold of 350. This threshold value provides an optimal output when performing the focus filtering method on the dataset. Images with $U{\left(I\right)}_{var}$ values less than 350 are considered blurry. To determine this threshold, we utilized a basic grid-search algorithm with numbers ranging from 0 to 525 and increasing by 175 each time. As this technique involved human determination of the threshold value, we assessed the results qualitatively. As a last step, we checked for possible outliers by looking at the quality of the unfocused X-rays images. We found 253, 125, 196, 86, and 51 outlier images respectively for normal, doubtful, minimal, moderate, and severe KOA, respectively, which were all put back into the focused sets. So, we have manually eliminated images from the merged dataset. After eliminating blurry images, a total of 7,949 images, including 3,080 normal images, 1,450 images of doubtful KOA, 2,059 images of minimal KOA, 1,080 images of moderate KOA and 280 images of severe KOA, remain. Fig. 9 shows some of the outlier images of the dataset.

#### 5.2.4 Noise reduction

While the contrast of the images is improved, minor noise is still present in the images might reduce the model's accuracy. To denoise the images, we follow three steps which are explained below: Otsu threshold to converting a grayscale image into a binary image, inverting the image, and morphological closing.

##### 5.2.4.1 Otsu thresholding

The Otsu thresholding technique, which employs a non-linear operation, is extensively used to convert an image from grayscale to binary format [53]. So, following the below process, this algorithm converts an image to binary format according to the intensity level of the input image.

If intensity [pixel] > particular threshold, the resultant pixel = 0 (black)

Else if intensity [pixel]≤particular threshold, the equivalent output pixel = 1 (white)

**Fig. 10:**
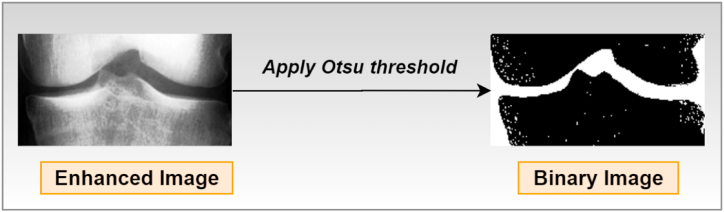
Output of the Otsu thresholding method.

So, Fig. 10 demonstrates that the objective of using Otsu thresholding to convert the image to binary has been accomplished.

##### 5.2.4.2 Inverting the image

**Fig. 11:**
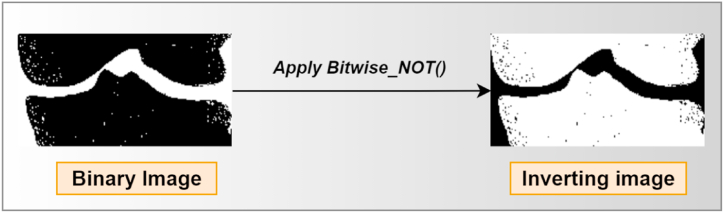
Output of the Bitwise_NOT method.

After generating the binary image, the image is inverted, which means that all the white pixels become black and all the black pixels become white. The background and the joint gap are now black and the bones white. Fig. 11 shows the inverted image.

##### 5.2.4.3 Morphological closing

**Fig. 12:**
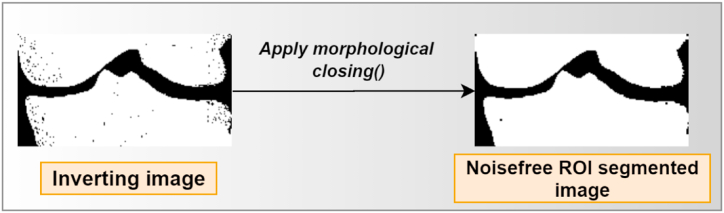
Output of the morphological closing method.

The traditional morphological closing technique can only eliminate regional details in high and low grayscale pixels. In the process of smoothing images, closing through by reconstruction can erase or keep regional features smaller than the present size in high and low-gray-scale regions [54]. The morphological operation first stretches an image, and then it erodes the stretched image. Both of these steps use the same structuring element. It can be used to fill in small gaps in an image while maintaining the size and shape of larger gaps and objects [55]. In the last phase of the processing system, we employed a morphological closing operation on the inverted pictures as the source image for producing a smooth X-ray image. Fig. 12 shows the output of the morphological closing method.

A black-and-white ROI is taken from each picture after completing these processes. Features will be extracted, utilizing these segmented images.

### 5.3 Proposed approach

#### 5.3.1 Preparing the dataset for feature extraction

**Fig. 13:**
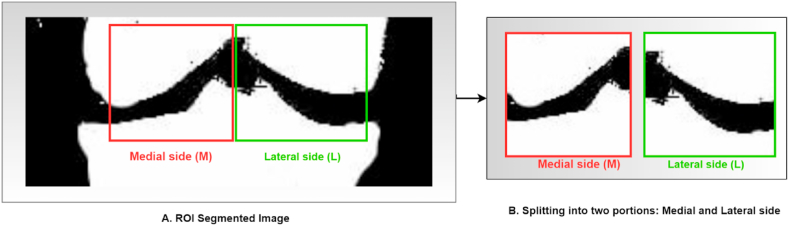
Output of the separation into medial and lateral sides.

A knee diagram has two parts: medial (inner) side and lateral (outer) side. During the KOA phase, these two parts are not affected in the same way. Therefore, we try to identify the affected area of the two compartments. Since the medial compartment of the knee is most affected compared to the lateral compartment, the segmented images were divided into medial and lateral segments. We prepared our dataset using these segmented images for feature extraction. To downsize the images, we employed different pixel sizes, including 70 × 50, 60 × 80, 50 × 80, etc., However, after that, each image pixel is downsized to 80 × 60 from a 200 × 100 image size as it provides an accurate area on the medial and lateral sides of an X-ray image. As a result, 15,838 images were generated from the previous 7,919 images. Fig. 13 illustrates the process.

#### 5.3.2 Feature extraction

The Feature extraction procedure is essential for classification. Generally, a collection of images can be categorized based on their most distinguishing characteristics. These can be attained by implementing feature extraction methods, which provide parameter values based on which the system can make a decision [56]. Six different feature sets are introduced: morphological features (set1) [57,58], GLCM features (set 2) [59], statistical features (set 3) [57], texture features (set 4) [60], LBP features (set5) [61], and a proposed feature (set 6). The feature extraction methods were employed to the 15,838 images (after splitting them into the medial and lateral sides). Table 5 describes the first five sets of features.

**Table 5:** Five sets of extracted geometrical features from ROI images.

Category	Feature name	Feature descriptions
**Set1:** **Morphological features**	Kurtosis	The density distribution of pixels
	Skewness	The degree of symmetry in the image's pixel distribution.
	Extent	The proportion of the total area of the ROI to the total area of the convex hull.
	Solidity	Using the pixels that make up the Convex Hull to contrast object regions with its Convex Hull.
	Circularity	The measurement of the ROI's roundness
	Major axis length	Calculate the longest length of the ROI object
	Minor axis length	Calculate the minimum length of the ROI object
	Equivalent diameter	This is the radius of a circle with the same circumference as the ROI region.
**Set 2:** **GLCM features**	Energy	The square root of an angular second moment is used to calculate energy. When the window is neatly arranged, energy has a larger value.
	Contrast	Contrast is a unique GLCM moment that is used to quantify the spatial frequency of an image. It's calculated by taking the range from the highest and lowest neighboring pixel values.
	Dissimilarity	Dissimilarity is a linear way to measure the differences between parts of an image.
	Homogeneity	It assesses image homogeneity by assuming bigger values for smaller variances in gray tone within-pair components. Homogeneity in the GLCM is particularly sensitive to the presence of near diagonal components.
	correlation	It is a measure of the linear relationship between the gray tones of the image.
	Entropy	It evaluates the randomness of intensity levels in the neighborhood.
**Set 3:** **Statistical features**	Mean	The sum of all pixels divided by the total number of pixels
	Standard deviation	The measurement of dispersion in the image's gray intensity level.
**Set 4:** **Texture features**	Texture Energy	It shows how rough the surface is in the defect image.
	Texture Entropy	This denotes the textural complexity of the fault image
**Set 5:** **LBP features**	LBP Energy	The LBP features are produced by contrasting the central pixel with its surroundings in a limited area of the image. These features define the image local texture properties and provide important advantages, including rotation and gray invariance.
	LBP Entropy	

#### 5.3.3 Proposed features (set 6)

In addition to the features mentioned above, we propose several features specific to this classification problem, including the distance: $d_{M1,}d_{M2,}d_{M3,}$ $d_{L1,}d_{L2}$ and $d_{L3},\text{medial}$ peak value, lateral peak value, medial area, lateral area, medial gradient and lateral gradient. These features represent differences in the distance between the femur and the tibia. Details are given below.

##### 5.3.3.1 Distance

**Fig. 14:**
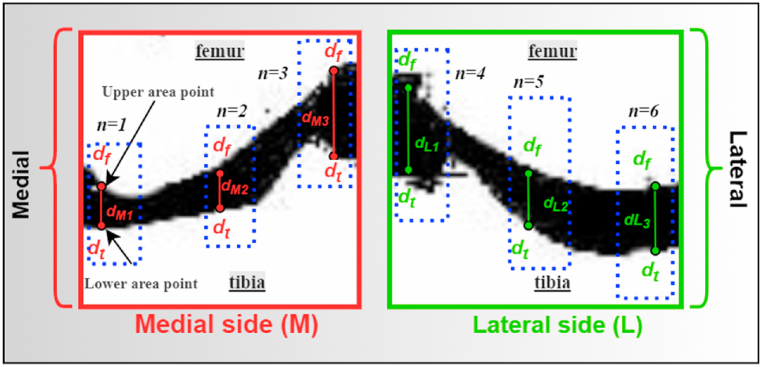
Distance measurement process for the medial and lateral side of the X-ray image.

To compute the distances between the bones, it is crucial to determine several upper and lower points for the area between the bones. In this regard, we assume three lower area points by setting the pixels width values respectively as 10, 40, and 70 (left to right), keeping the distance between them at 30. After trying with different sets of points, such as (20,50,80), (15,35,55), etc., we didn't acquire the desired results. Then, we set an ideal range of (10, 40, 70) to point to the top and bottom of the femur and tibia in the X-ray image. We set them for the tibia bone (in both the medial and lateral images), while x coordinates of the three upper area points (on the femur) are automatically determined based by the corresponding x-coordinates on the tibia bone. The main purpose is to calculate the joint space between the femur and the tibia. We, therefore, compute six distances: $d_{M1,}d_{M2}$ and $d_{M3}$ for the medial side image and $d_{L1,}d_{L2}$ and $d_{L3}$ for the lateral side images, as shown in Fig. 14.

This distance is computed by counting the number of pixels there are between two corresponding edge pixels on each side of the knee in the same column. Let $d_{f}$ and $d_{t}$ denote the corresponding edge pixels of the femur and tibia, respectively, as shown in Fig. 14. The vertical distance $d_{i}$ between the corresponding ith edge pixels of edges $d_{f}$ and $d_{t}$ is calculated as,

(13) $$d_{i}=\left|d_{f,i}^{y}-d_{t,i}^{y}\right|$$

where if the number of columns in the image is *n,* 1≤i≤n, $d_{f,i}^{y}$ and $d_{t,i}^{y}$ indicates the y-coordinate of the ith edge pixel of $d_{f}$ and $d_{t}$, respectively. Fig. 14 depicts the vertical distance between two corresponding edge pixels on both sides of an x-ray image using red and green lines. The distance between the femur and tibia for the medial and lateral sides of the knees is determined by taking the value of $d_{i}$. The distance in equation (13) is in pixels, nevertheless, JSW must be expressed in distance units, i.e., millimeters, in order to be used as an ion of osteoarthritis. Therefore, $d_{i}$ is converted to millimeters using PNG image properties, i.e., pixel-per-inch (ppi). Moreover, the joint space width for the lateral side is calculated analogously.

##### 5.3.3.2 Area (medial area and lateral area)

**Fig. 15:**
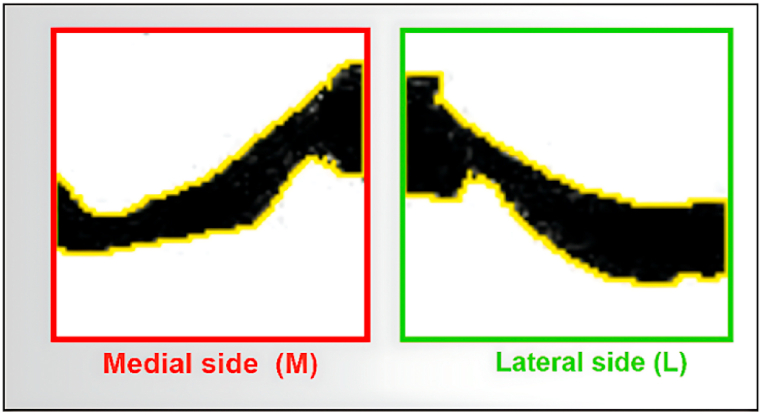
Area for the medial and lateral side of the X-ray image.

The area is calculated for both the medial and lateral images. The total of all pixels of the segmented gap between the femur and tibia of the medial and the lateral sides are considered area. Fig. 15 illustrates the area of the medial and lateral image.

##### 5.3.3.3 Peak value (medial peak value and lateral peak value)

**Fig. 16:**
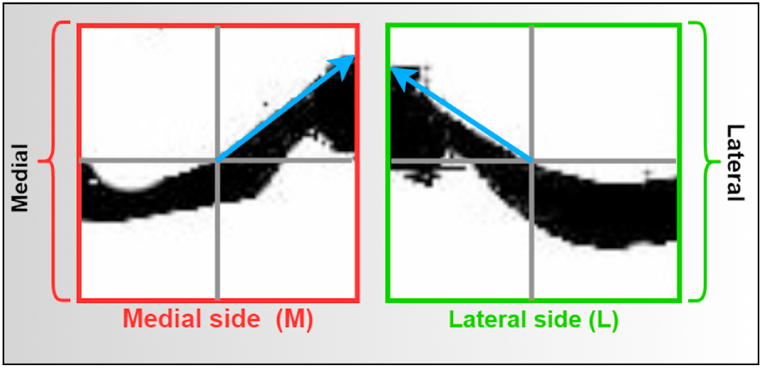
Measurement of the peak angle value for the medial and lateral side.

The peak value of an X-ray image's medial and lateral sides is measured to assess the angle. A straight line (X-axis) is drawn horizontally across the middle of the medial and lateral side image, as illustrated in Fig. 16. Another straight line (Y-axis) is drawn vertically (middle of the medial and lateral side) at a 90-degree angle with the X-axis. Then the peak value of the knee gap is illustrated as the blue line in Fig. 16. Peak values based on angles help to understand the maximum gap in the X-ray image, which helps to determine the grade. For normal grades, the maximum values of the medial and lateral side images are similar, while the value may be different for other grades.

##### 5.3.3.4 Gradient feature (medial gradient and lateral gradient)

**Fig. 17:**
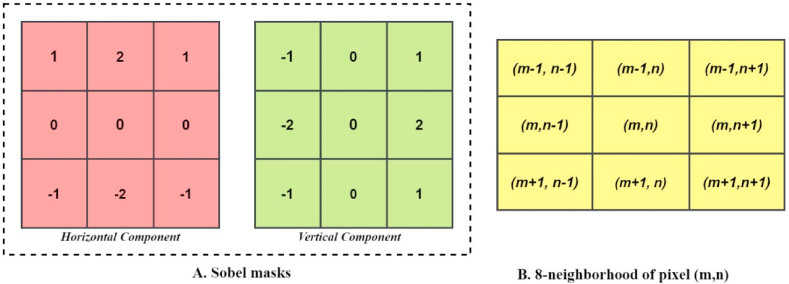
A. Sobel masks and B. 8 neighborhoods of (m,n) pixel.

This is a method that is utilized for extracting features from images. The gradient value is calculated for both images. It is a vector quantity that has both a magnitude and a direction and can be found by taking the derivatives of the function in both the horizontal and the vertical directions. The gradient operator creates a 2D gradient vector at each image point for the image. Its magnitude reflects the rate of change in the direction of the maximal rise in intensity. In this case, we utilized the Sobel operator to determine the Gradient Vector ${\left[G_{x},G_{y}\right]}^{T}$, where $G_{x}$, and $G_{y}$ denote the horizontal and vertical gradient components respectively. Fig. 17 (A) represents the horizontal and vertical masks of the Sobel operator used to calculate the horizontal and vertical components of the gradient.

I denotes the input image with A×,B at each pixel (m,n), where m=1 to A, n=1 to B. An 8-pixel neighborhood is created which is then further convolved with these Sobel masks to determine $G_{x}$, and $G_{y}$. The eight neighborhoods of pixel (m,n) are shown in Fig. 17 (B). $G_{x}$, and $G_{y}$ can be computed using the following equations no (14–17):

(14) $$G_{x}\left(x,y\right)=I\left(m-1,n-1\right)+2\times I\left(m-1,n\right)+I\left(m-1,n+1\right)-I\left(m+1,n-1\right)-2\times I\left(m+1,n\right)-I\left(m+1,n+1\right)$$

(15) $$G_{y}\left(x,y\right)=I\left(m-1,n-1\right)+2\times I\left(m,n-1\right)+I\left(m+1,n-1\right)-I\left(m-1,n+1\right)-2\times I\left(m,n+1\right)-I\left(m+1,n+1\right)$$

The Gradient strength and direction can be computed from the gradient vector ${\left[G_{x},G_{y}\right]}^{T}$ as:

(16) $$Gradient\;Magnitude=\left|G\left(m,n\right)\right|=\sqrt{{\left(G_{x}\left(m,n\right)\right)}^{2}+{\left(G_{y}\left(m,n\right)\right)}^{2}}$$

(17) $$theta\left(m,n\right)=\tan^{-1}\left\{\frac{G_{y}\left(m,n\right)}{G_{x}\left(m,n\right)}\right\}$$

**Fig. 18:**
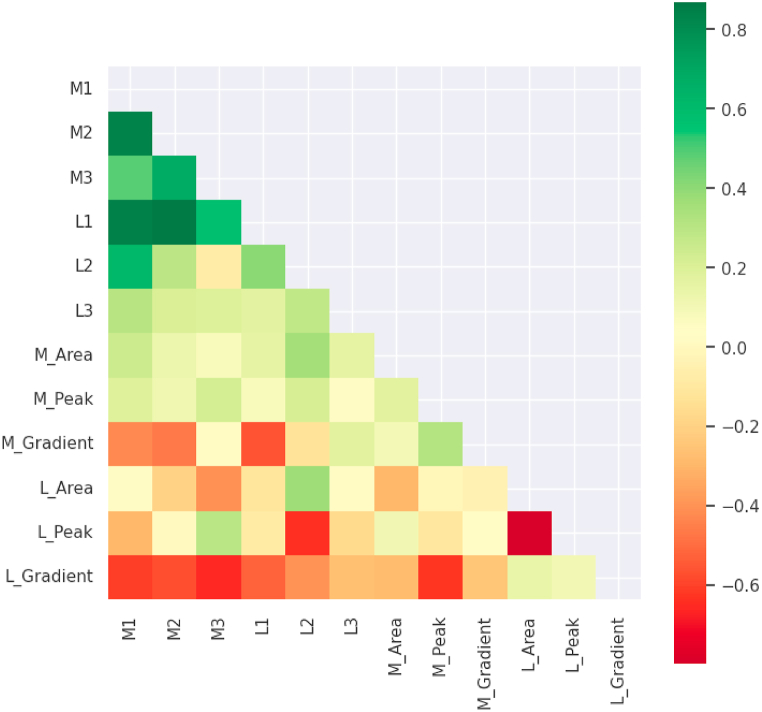
Heatmap showing correlations among all the proposed features.

Fig. 18 shows a heatmap of the correlations between all features. All coloured cells illustrate a relationship between two features, the correlation values. The strength of the relationship is shown by the colour of the cell. When the value is less than zero, there is a negative correlation; when it is equal to zero, there is no correlation [62]. Fig. 18 demonstrates that the M1, M2, M3, L1, L2, and L3 features have a strong correlation with the M1, M2, M3, L1, L2, and L3 features while there is a weak correlation between L2 and M3. Furthermore, a noticeable correlation between L_Area and M_Area can be observed. In contrast, a weak correlation can be observed between L_Peak and L_Area. The importance of these features can be determined after experimenting with different ML models and observing the results of different feature selection techniques.

### 5.4 Machine learning

In this work, five ML algorithms were used: Decision Tree (DT), Gradient Boosting (GB), KNN, XGB, and RF. These five models were used to determine the optimal features and classify KOA based on six different feature sets.

### 5.5 Training ML algorithms

**Fig. 19:**
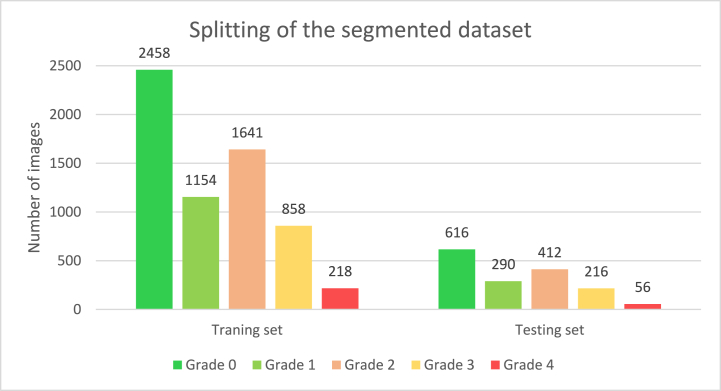
Class distribution of test and train set after splitting the segmented dataset.

In this step, we split our six distinct numerical feature datasets into a training and test set with a ratio of 80:20. The main goal of using an 80:20 ratio was to train our model with a large amount of data so that the model can learn from a variety of data. Fig. 19 shows how the segmented X-ray images were distributed across all five classes in the training and testing sets for the six sets of features. The training set consists of 6,329 segmented X-ray images and the test set consists of 1,590 segmented X-ray images. Thirty images were kept separate for further testing purposes to assess our proposed approach.

## 6 Results and discussion

This section explores the performance of five ML models for classifying knee OA using different feature sets. In addition, various feature selection techniques are applied to determine the features that should be considered to classify the KOA grades of patients. We also propose a decision tree strategy that helps to classify the stages based on the optimal features.

### 6.1 Evaluation metrics

To assess the performance of the ML models, several statistical metrics [58,63] such as accuracy (ACC), recall, precision, specificity, and F1-score are computed following equations no 18 to 22. All of these variables are calculated using the confusion matrix's True Positive (TP), True Negative (TN), False Positive (FP), and False Negative (FN) values.

(18) $$\text{ACC}=\frac{\text{TP}+\text{TN}}{\text{TP}+\text{TN}+\text{FP}+\text{FN}}$$

(19) $$\text{Recall}=\frac{\text{TP}}{\text{TP}+\text{FN}}$$

(20) $$\text{Precision}=\frac{\text{TP}}{\text{TP}+\text{FP}}$$

(21) $$\text{Specificity}=\frac{\text{TN}}{\text{TN}+\text{FP}}$$

(22) $$F_{1}=2\frac{\text{precision}*\text{recall}}{\text{precision}+\text{recall}}$$

### 6.2 Accuracy comparison of different ML models based on six distinct feature sets

This section describes the accuracy comparison of the five different ML models. We employed six different features sets: Set1: morphological features (16), Set2: GLCM features (12), Set3: statistical features (4), Set 4: texture features (4), Set 5: LBP features (4), and Set 6: proposed features (12) to these five models. Table 6 shows that the XGB model obtained the highest accuracy of 96.64 % utilizing the proposed 12 features. The RF model acquired the second-highest accuracy of 96.48 %, and the GB model achieved the third-highest accuracy of 96.01 % using the proposed features (Set 6). The XGB model acquired the highest accuracy of 85.61 % for the other feature sets. The RF and GB model obtained the second and the third highest accuracy of 84.79 % and 82.23 % respectively for the other features. In contrast, GB acquired the lowest accuracy of 52.06 % accuracy using the texture features. In conclusion, the XGB model combined with our proposed 12 features (feature set 6) performed very well in knee OA classifications compared to the other feature sets. Consequently, we choose the XGB ML model and feature set 6 for further processing.

**Table 6:** Accuracy of five different ML models based on six distinct feature sets.

Sets of features	Five ML Models' Accuracy				
	DT	RF	KNN	XGB	GB
Set1: Morphological features (16)	81.29 %	84.79 %	79.03 %	85.61 %	82.23 %
Set2: GLCM features (12)	76.92 %	78.08 %	73.37 %	78.94 %	77.08 %
Set3: Statistical features (4)	56.26 %	57.35 %	53.44 %	58.43 %	59.03 %
Set4: Texture features (4)	64.93 %	66.28 %	59.12 %	71.53 %	52.06 %
Set5: LBP features (4)	71.18 %	74.56 %	69.88 %	78.37 %	70.38 %
Set6: Proposed features (12)	94.73 %	96.48 %	95.35 %	**96.64 %**	96.01 %

**Fig. 20:**
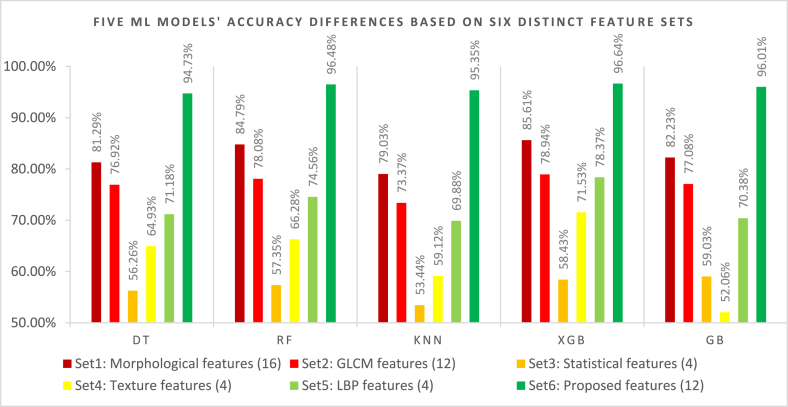
Five ML models' accuracy comparison based on six different feature sets.

Fig. 20 depicts the graphical representation of the accuracies of five ML models based on six different types of feature sets. From Fig. 20, it can be seen that all the models achieved the best results using only our proposed twelve features. The XGB model achieved the highest accuracy of 96.64 %. Also, the lowest accuracy using our proposed features was 94.73 %. So, the accuracy ranges from 94.73 % to 96.64 %. In contrast, the models performed poorly by achieving unsatisfactory accuracy with other feature sets. So, we chose the proposed twelve features set to continue further experiments.

### 6.3 Feature selection

Finding the optimal selection of features in ML is essential since using those can assist in minimizing time complexity. Various feature selection techniques are used in this work, including univariate features, correlation matrix, the principal component analysis (PCA) method, and the wrapper method. The univariate feature selection methods disregard the mutual information between features with specific independence or orthogonality assumptions. Some features may contribute little to classification (these would be filtered out using univariate methods) and some may improve performance when combined with other features [64]. The correlation-based feature selection approach can eliminate a greater number of redundant and irrelevant features and offers slightly better performance and less complexity than previous strategies [65]. In contrast, PCA is the most popular linear dimensionality reduction method and has been extensively used to analyze datasets across all scientific domains. PCA aims to preserve all pertinent linear structures when mapping or embedding data points from a high-dimensional space to a low-dimensional space. Moreover, it can determine the key variables that explain the differences and can be used to facilitate the analysis and visualization of high-dimensional datasets without losing any essential information [66]. The wrapper method interacts with classifiers for feature selection. It is a more exhaustive search of features that takes feature dependencies into account. Moreover, it has better generalization than the filter approach [67]. The results of these feature selection techniques are provided in the result section.

**Fig. 21:**
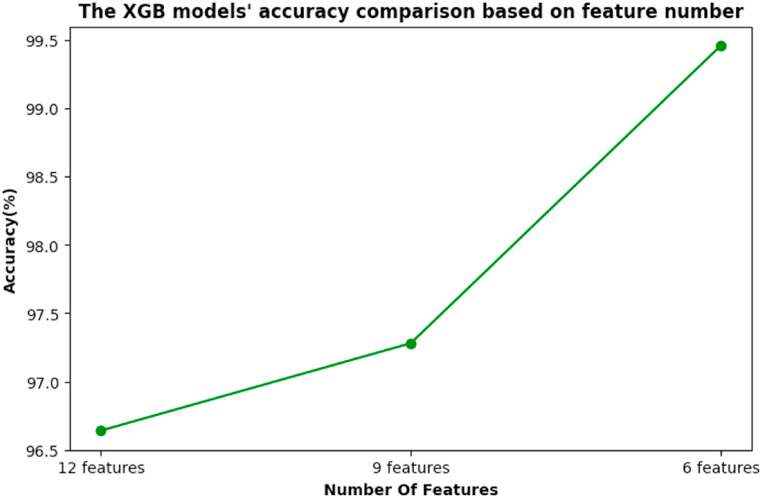
Accuracy comparison of the XGB model based on feature number.

The aim is to determine the optimal selection of features from the proposed features. Table 7 shows the performance of the XGB model combined with different feature selection techniques for our proposed features. For feature selection, correlation matrix, wrapper method, PCA, and univariate feature selection methods were employed with the twelve proposed features. Table 7 shows that the correlation feature selection technique provides nine features, M1, M2, M3, L1, L2, L3, M_Area, M_Peak, and L_Area, resulting in a 97.28 % accuracy and 96.67 % F1-score for the XGB model. The wrapper feature selection method, PCA, and univariate feature selection approach provide the same features: M1, M2, M3, L1, L2, and L3. The XGB models' accuracy and the F1-score were 99.46 % and 99.1 % respectively, for these features. Since the majority of the feature selection techniques provide six features, these may be considered as the optimal features in knee OA diagnosis. It is also notable that the best ML model's (XGB) performance improved with feature selection. For a better understanding, a visual representation is given in Fig. 21.

**Table 7:** Performance evaluation of different feature selection methods on XGB ML model.

Feature Selection	Feature Number	Feature Name	Accuracy	F1 Score
All features	12	M1, M2, M3, L1,L2,L3,M_Area, M_Peak,M_Gradient, L_Area, L_Peak,L_Gradient	96.64 %	95.92 %
Correlation Matrix	9	M1, M2, M3, L1, L2,L3,M_Area, M_Peak, L_Area	97.28 %	96.67 %
Wrapper Method	6	M1, M2, M3, L1, L2,L3	99.46 %	99.1 %
PCA	6	M1, M2, M3, L1, L2, L3	99.46 %	99.1 %
Univariate Feature	6	M1, M2,M3,L1,L2,L3	99.46 %	99.1 %

### 6.4 Analysis for the six optimal features

**Fig. 22:**
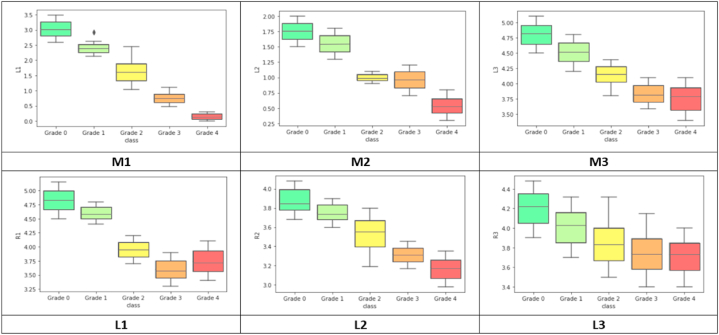
Box plots of optimal six features.

The distribution of data can be visualized with box plots. A box plot helps to understand how the data are distributed and represents the highest, lowest, and median values. This study evaluated the values of the six optimal feature values for every disease class. The six optimal feature values corresponding to the KOA grades are presented using box plots in Fig. 22.

**Fig. 23:**
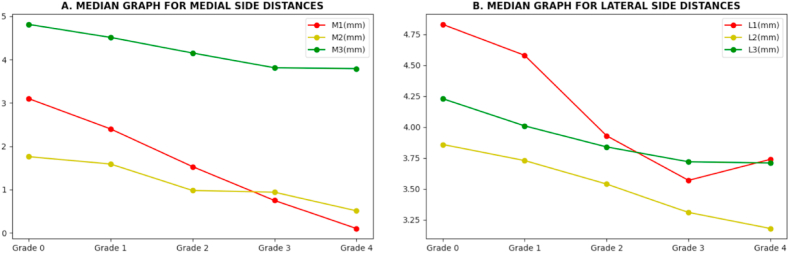
Median graph for six distance values (optimal features) of the medial (A) and lateral (B) sides.

Table 8 presents the standard distance range of the six features corresponding to the KOA grades. All the values are in millimeters. For the normal class (Grade 0), the standard median values of M1, M2, and M3 are 3.10 mm, 1.76 mm, and 4.83 mm, respectively. For Grade 1, the medial side distances of M1, M2, and M3 values are reduced by 0.7 mm, 0.17 mm, and 0.3 mm and the lateral side distance values for L1, L2, and L3 are reduced by 0.25 mm, 0.13 mm, and 0.22 mm compared to Grade 0. Comparing Grade 2 to the normal class, the median values of the M1, M2, and M3 are reduced by 1.57 mm, 0.78 mm, and 0.66 mm, while the L1, L2, and L3 values are reduced by 0.9 mm, 0.32 mm, and 0.39 mm. Likewise, for Grade 3, the differences compared to Grade 0 of M1, M2, M3, L1, L2, and L3 are 2.35 mm, 0.82 mm, 1.00 mm, 1.26 mm,0.55 mm, and 0.51 mm. For grade 4, the differences in M1, M2, M3, L1, L2, and L3 are 3.00 mm, 1.25 mm, 1.02 mm, 1.09 mm, 0.68 mm, and 0.52 mm compared to normal. Here, we can see that the normal grade distance values are higher than other grades. However, Fig. 23 shows the median value graph for both medial and lateral-side distance values for better understanding. Thus, it can be concluded that the joint gap distances progressively decrease as the gradient increases.

**Table 8:** The standard distance ranges for the six optimal features based on the KOA grade.

Grade name	Median value (Lower quartile -Upper quartile) mm					
	M1	M2	M3	L1	L2	L3
**Grade 0**	3.10(2.78–3.24)	1.76(1.63–1.88)	4.81(4.64–4.93)	4.83(4.66–4.99)	3.86(3.78–3.99)	4.23(4.07–4.35)
**Grade 1**	2.40(2.29–2.52)	1.59(1.38–1.69)	4.51(4.37–4.67)	4.58(4.51–4.70)	3.73(3.68–3.85)	4.01(3.86–4.15)
**Grade 2**	1.53(1.35–1.80)	0.98(0.91–1.08)	4.15(4.06–4.28)	3.93(3.84–4.08)	3.54(3.40–3.67)	3.84(3.68–3.99)
**Grade 3**	0.75(0.60–0.84)	0.94(0.81–1.11)	3.81(3.68–3.96)	3.57(3.46–3.77)	3.31(3.26–3.39)	3.72(3.56–3.89)
**Grade 4**	0.10(0.08–0.27)	0.51(0.39–0.63)	3.79(3.57–3.92)	3.74(3.59–3.92)	3.18(3.06–3.27)	3.71(3.54–3.84)

### 6.5 Performance analysis of the five ML models utilizing optimal six features

Table 9 lists the accuracy, precision, recall, specificity, and F1-score for four ML models using the optimal six-distance features. We previously obtained better performance with the XGB model, where the precision, recall, specificity, and F1-score were 99.25 %, 99.43 %, 99.17 %, and 99.1 %, respectively. The RF model acquired the second-highest accuracy of 99.17 %, a precision of 99.06 %, a recall of 98.11 %, a specificity of 98.97 %, and an F1-score of 98.67 %. The DT model acquired the third-highest accuracy of 98.72 %, while the precision, recall, specificity, and F1 score were 97.88 %, 97.99 %, 97.79 %, and 98.17 % respectively. The GB and the KNN model acquired values for the accuracy and other performance metrics which were the closest to the best model (XGB). Table 9 demonstrates that for the four ML models' previously described, the performance was improved by using the six optimal features rather than working with another number of features.

**Table 9:** Performance analysis of the five ML models with 6 features.

Model Name	Accuracy	Precision	Recall	Specificity	F1 Score
DT	98.72 %	97.88 %	97.99 %	97.79 %	98.17 %
RF	99.17 %	99.06 %	98.11 %	98.97 %	98.67 %
KNN	98.03 %	97.05 %	97.09 %	97.68 %	98.12 %
GB	98.21 %	97.37 %	97.91 %	97.68 %	98.14 %
XGB	**99.46 %**	99.25 %	99.43 %	99.17 %	99.1 %

### 6.6 Pair wise statistical analysis of optimal six features

Several pairwise statistical values, including F-value, Z-test value, and P- value, are computed for the six optimal features. The F-test is utilized to compare the variance of at least two groups. It is a ratio of two variance measures to perform a hypotheses test. A z-test is used to assess if two two-group means are distinct when the sample size is large, and the variances are known. The p-value is a statistical measure used to evaluate the significance of the correlation between two variables. A p-value less than 0.05 denotes statistical significance, and less than 0.01 denotes high statistical significance. Table 10 shows that all the p values for all features are less than 0.05, which means all features are statistically significant in this study. A z-score greater than 1.96 (or less than −1.96) indicates a statistically significant difference at the 95 % confidence level when comparing the sample mean to the population mean. Since the maximum z-test values are greater than 1.96, these features are significant with a large confidence level.

**Table 10:** Analyzing the best six features via pairwise statistics.

Features name Vs Class	F-value	Z-test value	P-value
M1 vs Class	4.66	2.37	0.0089
M2 vs Class	5.43	2.43	0.0075
M3 vs Class	3.32	2.11	0.0174
L1 vs Class	3.91	1.96	0.0249
L2 vs Class	2.19	1.79	0.0367
L3 vs Class	1.68	1.73	0.0418

## 7 Further discussion

### 7.1 Experimentation with separate images

As previously stated, 30 X-ray images were kept separate in this study and were not included in any train or test scenario. To evaluate the grade of the images, these 30 images were individually prepared following the ROI segmentation process resulting in 60 images after division into medial and lateral -side images. The six distance features were extracted from each image and compared to the standard range of the six distance features, corresponding to the five KOA grades. If these newly extracted features meet the characteristics of a class (for five or more features), the image might be classified to the corresponding KOA grade. The proposed approach is described in pseudocode 1. Table 11 presents the six feature values and the test results of these 30 images. The features were compared to the standard distance range of five grades shown in Table 8.

**Figure:**
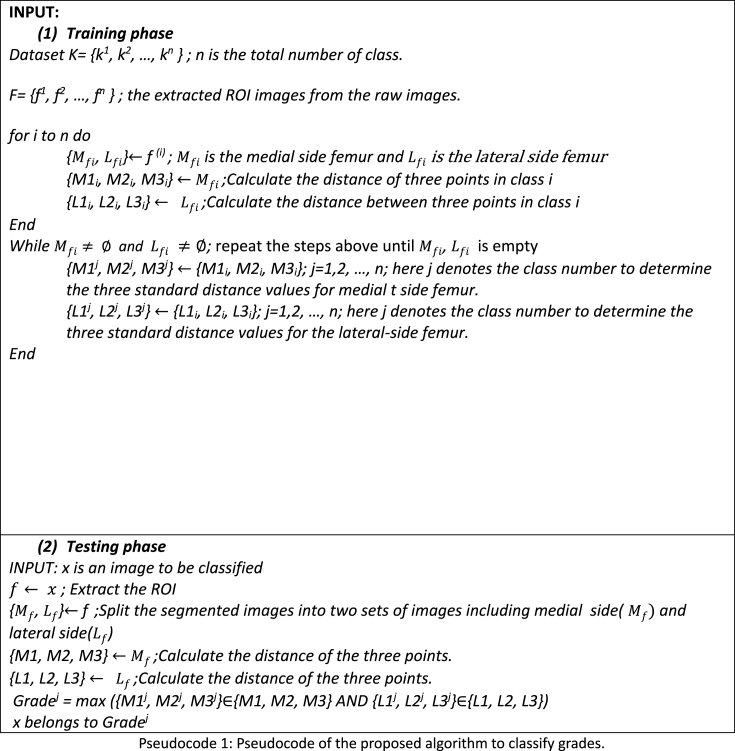

**Table 11:**
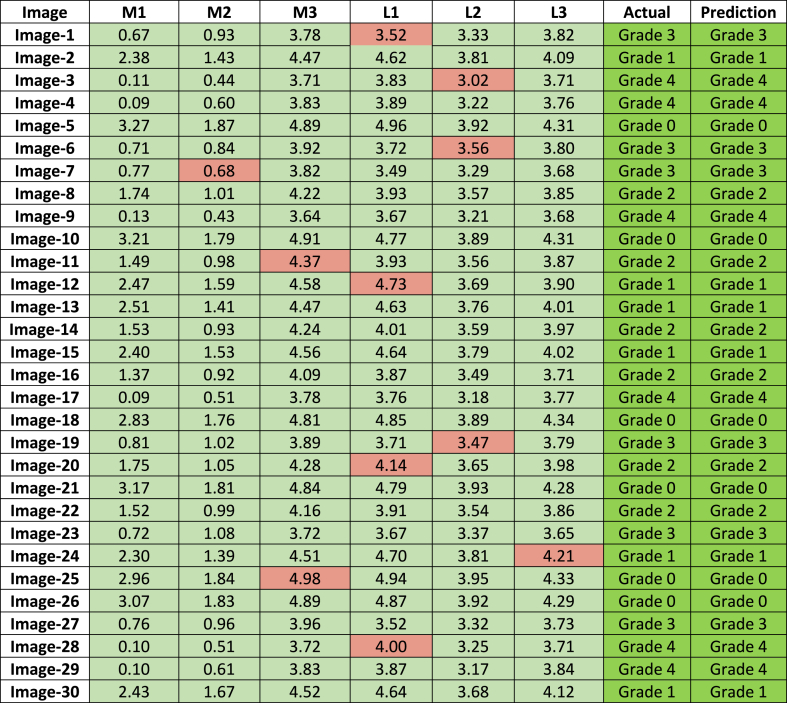
Classification results of 30 distinct X-ray images according to the proposed approach.

**Table 12:** Accuracy comparison with existing literature.

Paper	Name of the dataset and number of X-ray image	Segmentation Features and Models name	Class name	Highest accuracy and optimal feature number	Limitations
[21]	Collected from Osteoarthritis Initiative (OAI) dataset: 1,000 images	Segmentation: Not applied Features: **Set1.** InceptionV3, **Set2**.Xception, and **Set3**. DenseNet201 Model: SVM, LR and RF	Healthy, Doubtful, Minimal, Moderate, and Severe.	Accuracy: 71.33 % (DenseNet201-SVM) Feature num: Not given. (Surely more than 6)	1.No experimentation with a combined dataset 2. No experimentation with various ML models 3.Lack of image segmentation 4. Utilized more than 6 features 5. Lack of using large datahub. 6. Lower performance in multi-class 7. Lack of introducing decision making system
[5]	Collected data: 200 images	Segmentation: Active contour segmentation method Features: Haralick (4), Statistical (2), First-four moments (4), Texture (1) and Shapes (7) Model: Random Forest (RF)	Normal and Affected	Accuracy: 87.92 % (RF) Feature num:18 (merged feature set)	1. No experimentation with a combined dataset 2. Lack of using large data-hub for five grades 3. No experimentation with various ML models 4. Utilized more than 6 features 5. Lower performance in multi-class 6. Lack of introducing decision making system
[22]	Collected data: 350 images	Segmentation: Local center of mass (LCM) Features: Histogram Based (mean, variance, skewness, kurtosis, energy, and contrast) GLCM Based Features (correlation, entropy, homogeneity) Model: CNN	Healthy, Doubtful, Minimal, Moderate, and Severe.	Accuracy: 93.2 % (CNN) Feature num:9	1. No experimentation with a combined dataset 2. No experimentation with various transfer learning (TL) models 3. Utilized more than 6 features 4. Lack of using large data-hub for five grades 5. Lower performance in multi-class 6. Lack of introducing decision making system
[23]	Collected data: 50 images	Segmentation: Only cropped Features: Euclidean distance, Manhattan distance, Cosine, and Correlation Model: K-nearest neighbor (KNN)	Normal, Abnormal, Medium, and Worst	Accuracy: 95.24 % (Normal or Abnormal), 97.37 % (Medium or Worst) Feature num: 4	1.No experimentation with a combined dataset 2. No experimentation with various ML models 3.Lack of segmentation 4. Lack of using large data-hub for five grades 5. Lower performance in multi-class classification 6. Lack of introducing decision making system
[24]	Mendeley IV dataset: 2,000 images	Segmentation: Only cropped Features: •CNN + HOG •CNN + LBP (Combination of high- and low-level features) Model: KNN + HOG + CNN	Healthy, Doubtful, Minimal, Moderate, and Severe.	Accuracy: 97 % (KNN + HOG + CNN) Feature num: More than 6	1.No experimentation with a combined dataset 2.Absence of proper ROI segmentation 3. Utilized more than 6 features 4. Lower performance in multi-class 5. Lack of using large data-hub for five grades 6. Lack of introducing decision making system
[25]	Mendeley IV dataset**:** 2,000 images OAI: 5045 (Grade 0) +3,967 (Grade I-IV)	Segmentation: Not clear Features: Using CenterNet method Model: DenseNet201	Healthy, Doubtful, Minimal, Moderate, and Severe.	Accuracy:99.14 % (Test accuracy),98.75 % (Cross-validation accuracy) Feature num: More than 6	1.No experimentation with a combined dataset 2. Lack of image processing 3. Utilized more than 6 features 4. Lack of introducing decision making system
[26]	Mendeley dataset (KOA severity grading dataset) 3,795 images	Segmentation: Not implemented (Image processing done) Features: CNN features using Darknet-53 and AlexNet Feature selection method: PCA Model: YOLOv2-ONNX	Healthy, Doubtful, Minimal, Moderate, and Severe.	Accuracy (mAP): 98 % Features num: More than 6	1.No experimentation with a combined dataset 2. No experimentation with various ML models 3.Lack of image segmentation 4. Utilized more than 6 features 5. Lack of using large data-hub for five grades 6. Lack of introducing decision making system
[28]	First dataset: MOST (3,206 images) Second dataset: OAI (4,796 images)	Segmentation: Not implemented Feature extraction: N/A Feature selection technique: N/A Model: Deep Siamese Convolutional Neural Network	Healthy, Doubtful, Minimal, Moderate, and Severe.	Accuracy: 66.7 % Features num: Deep features (more than 6)	1. Lack of image processing method 2.Lack of image segmentation method 3. Utilized more than 6 features (deep features) 4. Lower performance in multi-class
**Proposed work**	Dataset1: Knee Osteoarthritis Severity Grading dataset (8,260) Dataset2: CGMH Knee Osteoarthritis Images (400). **Total images: 8,660-> 7,919 (eliminate 711 images + keep separate 30 images)-> 15,838 images (After dividing into two groups: medial and lateral side)**	**Segmentation**: Proposed ROI segmentation method **Featuresets:** **Set1:** Morphological feature (16) **Set2:** GLCM feature (12) **Set3:** Statistical feature (4) **Set4:** Texture feature (4) **Set5:** LBP feature (4) **Set6:** Proposed feature (12) Proposed feature: *Distance features (M1, M2, M3, L1, L2, and L3), * Area features (medial and lateral -side area), *Peak value (medial and lateral peak value), and *Gradient features (medial and lateral gradient) **Model:** XGB, GB, DT, RF, KNN	**Healthy, Doubtful, Minimal, Moderate, and Severe**	**Accuracy: 99.64 % (XGB model)** **Feature num: 6** **M1, M2, M3, L1, L2, and L3)**	**1.Progression of the disease is not included.** **2.Experiment with real data is not included.** **3.Need to experiment with real time large data hub.**

According to Table 8, the standard lower and upper quartile range for Grade 0 is considered (2.78–3.24) for M1, (1.63–1.88) for M2, from (4.64–4.93) for M3, (4.66–4.99) for L1, (3.78–3.99) for L2, and (4.07–4.35) of L3. For Grade 1, it ranges from (2.29–2.52) for M1, from (1.38–1.69) for M2, from (4.37–4.67) for M3, from (4.51–4.70) for L1, from (3.68–3.85) for L2, and from (3.86–4.15) for L3. For Grade 2, from (1.35–1.80) for M1, from (0.91–1.08) for M2, (4.06–4.28) for L1, (3.40–3.67) for L2, and (3.68–3.99) for L3. Additionally, in the case of Grade 3, the ranges start from (0.60–0.84) for M1, from (0.81–1.11) for M2, from (3.68–3.96) for M3, from (3.46–3.77) for L1, (3.26–3.77) for L2, and from (3.56–3.89) for L3. Additionally, the ranges of the Grades start from (0.08–0.27) for, from (0.08–0.27) for M1, (0.39–0.63) for M2, from (3.57–3.82) for M3, from (3.59–3.92) for L1, from (3.06–3.27) for L2, and (3.54–3.84) for L3. So, Table 11 shows that for all 30 images at least five out of six features satisfy the requirements of being within the distance range for the same grade. As the extracted feature values are in the optimal range, the decision-making system is able to diagnose the grades corresponding to the results. Consequently, after examining the X-ray image findings, we can assert that our suggested technique with six distance characteristics can be used to identify the KOA grades.

### 7.2 Comparison with existing work

Table 12 provides a comparison between our proposed work and existing related work. The proposed method was compared with recent studies in KOA. Table 12 compares these previous studies with our proposed method based on the number of images, features number, proper segmentation process, and accuracy. As mentioned, knee ROI segmentation and feature extraction from the segmented images are essential for satisfactory accuracy. Consequently, several researchers have applied different ROI segmentation and feature extraction techniques. Teo et al. [21] worked with 1,000 X-ray images of five classes and did not apply any segmentation process. They generated a feature set using the DenseNet201 model and combined it with the SVM classifier. Their model acquired 71.33 % accuracy. They did not use any combined dataset and did not show any comparison with other ML models. Moreover, they generated more than 6 (our optimal features) features but failed to acquire satisfactory results. Shiva et al. [5] performed an active contour segmentation method on 200 images of normal and affected classes. After extracting 18 features, the RF classifier obtained 87.92 % accuracy. Though they experimented with only two classes, the accuracy was not satisfactory. Sikkandar et al. [22] collected 300 images of five grades, applied LCM segmentation, and extracted 9 features. Their CNN model acquired 93.2 % accuracy in multiclass classification. The core limitations were that they did not work with any combined dataset and did not show the comparison with other transfer learning models. Subramonium et al. [23] extracted four distance features from only 50 images in four classes without performing any segmentation. The KNN classifier achieved 97.3 % and 95.24 % accuracy for medium or worst class and normal or abnormal binary classification, respectively. As their model achieved 97.3 % accuracy for binary classification with 50 images, their accuracy might be degraded for multiclass classification. Similarly, Rabbia et al. [24] only cropped 2,000 X-ray images of five classes and extracted features by applying CNN and HOG. The KNN + HOG + CNN model acquired 97 % accuracy for more than six features. They might have used a larger dataset and segmentation in their work. In another case [25], researchers applied the CenterNet method, and their DenseNet201 model acquired 99.14 % and 98.75 % test and cross-validation accuracy, respectively. They did not use any merged dataset, and the number of features was more than six. Yunus et al. [26] also utilized only 3,795 images of five grades. At the same time, they performed only image processing methods, extracted CNN features using Darknet-53 and AlexNet models, and classified employing the YOLOv2-ONNX model that achieved a mAP of 98 %. Though their model acquired satisfactory accuracy in classification, the feature number was unclear, and did not use any large data hub. Moreover, there is a chance to degrade the models' performance using the large data. Nevertheless, the accuracy might be enhanced while performing optimal segmentation, feature extraction, and selection methods. In another work [28], researchers introduced the deep Siamese CNN model and obtained only 66.7 % accuracy, while they did not perform any segmentation, preprocessing, feature extraction, and selection techniques but models’ accuracy might be increased following the methods that we utilized in our work. Thus, the main limitations are the absence of segmentation, using a large and combined data hub of five grades, extracting optimal clinical features, selecting salient features, and introducing an automated decision-making system for KOA grade determination. As mentioned above, physical and mental health are interrelated, and any disease, including chronic pain, COVID-19, etc., can adversely affect both cases [[68], [69], [70]]. As we focused on KOA grade determination using an automated decision-making system, it may play an essential role in maintaining health status in the medical field. However, achieving good accuracy, finding the best process for segmenting ROIs, and extracting the prominent medical features from X-ray images are challenging, so complex methods are needed to achieve the best results. Our study aims to address these research gaps. We worked on a large dataset of 8,660 images of five classes to propose an appropriate ROI segmentation method. Feature extraction yielded six optimal distance features that could be effectively used to diagnose KOA. The XGB classifier performed with 99.46 % accuracy in multiclass classification using only six features. Furthermore, we proposed a decision-making strategy based on these optimal six features and successfully tested it with 30 isolated images for diagnosis.

## 8 Conclusion

This study addresses a multiclass classification problem for KOA using knee X-ray images by determining the optimal features and a decision-making system using these features. This work mainly aims to classify the KOA grades based on only the medical features (distance feature's value) without employing deep learning and machine learning frameworks. So, the key findings of this study are proposing an optimal segmentation method to segment the ROIs, introducing prominent medical features set, and making a decision system based on the extracted feature's value. Two distinct datasets: “Knee Osteoarthritis Severity Grading” and “CGMH knee osteoarthritis,” were combined, 711 outliers were eliminated from the combined dataset, and 30 images were kept apart for testing our proposed decision-making system. We proposed an effective ROI segmentation technique employing several image processing methods and segmented the knee ROI from 7,919 images, obtaining 15,838 images by splitting them into two groups: medial and lateral sides. After that, six different feature sets were generated, including set1: morphological features (kurtosis, skewness, extent, solidity, circularity, major axis length, minor axis length, and equivalent diameter), set 2: GLCM features (energy, contrast, dissimilarity, homogeneity), set 3: statistical features (mean and standard deviation), set 4: texture features (texture energy and texture entropy), set 5: LBP features (LBP energy and LBP entropy) and set 6: proposed features. The XGB classifier acquired the highest accuracy of 96.64 % with the proposed twelve features consisting of distance features (M1, M2, M3, L1, L2, and L3), area (medial and lateral), peak (medial and lateral peak value), and gradient (medial and lateral gradient value). After applying several feature selection techniques to the proposed features, six optimal distance features (M1, M2, M3, L1, L2, and L3) were identified, resulting in improved accuracy of the previous models. The XGB model acquired the highest accuracy of 99.46 % in multiclass classification. After exploring the six features, we determined the distance range of these features corresponding to the grades. We then proposed a decision-making system based on these six distance features (M1, M2, M3, L1, L2, and L3) and tested it with 30 isolated X-ray images. The decision system can effectively determine the KOA grades by comparing the range of the distance features with the range of these features corresponding to the grades. Table 8 and Fig. 23 represent the standard distance ranges and the median graph for six medial and lateral side distance values. It illustrates the changes in the feature values depending on the grades occurring in the KOA. As a result, obtaining distance feature values from images and feeding them in a decision system assists the professional in diagnosing the KOA without requiring any model or time complexity. The key benefit of this study is that anybody may use the suggested method to diagnose KOA quickly and effectively without encountering any complications. It will have a significant influence on KOA diagnosis in the medical field. Although the proposed framework and decision-making system are quite effective, some shortcomings have been noted that can be addressed in further research. So, in the future, we plan to extend the dataset with various knee X-ray images, testing our first proposed decision-making system, which could be tested with newly acquired and real-time data to evaluate our methods. Moreover, we may explore geometrical deep learning and graph neural networks to understand KOA's progression.

## 9 Implications

This study explores the practical implications of managing KOA diseases and proposes an automated decision-making strategy for healthcare professionals treating patients with this condition. With a comprehensive understanding of the underlying nature of knee osteoarthritis and the healthcare system, it becomes evident that this condition can cause many problems for people. It is a common and debilitating joint disorder affecting millions worldwide, causing pain, stiffness, and reduced mobility. Similar to other chronic health problems, KOA requires increased attention from healthcare professionals and policymakers to develop effective prevention, management, and treatment strategies. This study also emphasizes the importance of diagnosing KOA grades at an early stage, introducing an automated decision-making system based on extracting six prominent medical features from knee X-ray images. It presents a real-time application with isolated X-ray images for evaluating our proposed methods in terms of diagnosis. Deploying any deep learning and machine learning model generates different complexity, while our work can easily diagnose any grades without facing the complexity. Our work emphasizes the medical features relevant to the radiologist's knowledge in terms of diagnosis. Thus, giving patients the knowledge and skills to control their illness can enhance results and well-being. In addition, research suggests that extracting essential knee joint gaps (ROIs) is crucial, corresponding to the grades and analysis of the changes through the distance features of an image's medial and lateral portion to aid the radiologists in diagnosing. This study highlights the significance of measuring the distance features from the medial and lateral sides of the segmented ROIs. It introduces an automated decision-making system to manage this condition effectively.

## Data availability statement

Datasets used are publicly available:

• cgmh-oa.” Available: https://www.kaggle.com/datasets/tommyngx/cgmh-oa.

• Chen, Pingjun (2018), “Knee Osteoarthritis Severity Grading Dataset”, Mendeley Data, V1, https://doi.org/10.17632/56rmx5bjcr.1.

## Funding statement

This research does not have any external funding.

## CRediT authorship contribution statement

**Kaniz Fatema:** Conceptualization, Formal analysis, Writing – original draft. **Md Awlad Hossen Rony:** Formal analysis, Resources, Validation, Visualization. **Sami Azam:** Conceptualization, Formal analysis. **Md Saddam Hossain Mukta:** Data curation, Methodology. **Asif Karim:** Data curation, Resources, Writing – review & editing. **Md Zahid Hasan:** Resources, Validation. **Mirjam Jonkman:** Resources, Writing – original draft, Writing – review & editing.

## Declaration of competing interest

The authors declare that they have no known competing financial interests or personal relationships that could have appeared to influence the work reported in this paper.

## References

[1] Haq I., Murphy E., Dacre J. (2003). I haq, E murphy, J dacre. Postgrad. Med..

[2] Kubakaddi S., Urs N. (2019). Detection of Knee Osteoarthritis by Measuring the Joint Space Width in Knee X-ray Images l Jo l o f Ele c.

[3] Deokar D.D., Patil C.G. (2015). Effective feature extraction based automatic knee osteoarthritis detection and classification using neural network. International Journal of Engineering and Techniques.

[4] Turkiewicz A., Petersson I.F., Björk J., Hawker G., Dahlberg L.E., Lohmander L.S., Englund M. (2014). Current and future impact of osteoarthritis on health care: a population-based study with projections to year 2032. Osteoarthritis Cartilage.

[5] S., S., U., P., & R., R (2016). Detection of osteoarthritis using knee X-ray image analyses: a machine vision based approach. Int. J. Comput. Appl..

[6] Iorember P.T., Iormom B., Jato T.P., Abbas J. (2022). Understanding the bearable link between ecology and health outcomes: the criticality of human capital development and energy use. Heliyon.

[7] Abbas J., Wang D., Su Z., Ziapour A. (2021). The role of social media in the advent of COVID-19 pandemic: crisis management, mental health challenges and implications. Risk Manag. Healthc. Pol..

[8] Azadi N.A., Ziapour A., Lebni J.Y., Irandoost S.F., Chaboksavar F. (2021). The effect of education based on health belief model on promoting preventive behaviors of hypertensive disease in staff of the Iran University of Medical Sciences. Arch. Publ. Health.

[9] Gwilym S., Pollard T., Carr A. (2008). Understanding pain in osteoarthritis. J Bone Joint Surg Br.

[10] Ho-Pham L.T., Lai T.Q., Mai L.D., Doan M.C., Pham H.N., Nguyen T.V. (2014). Prevalence of radiographic osteoarthritis of the knee and its relationship to selfreported pain. PLoS One.

[11] Hannan M.T., Felson D.T., Pincus T. (2000). Analysis of the discordance between radiographic changes and knee pain in osteoarthritis of the knee. J. Rheumatol..

[12] Hani A.F.M., Malik A.S., Kumar D., Kamil R., Razak R., Kiflie A. (2011). Proceedings of the 2011 International Conference on Electrical Engineering and Informatics.

[13] Barr A.J., Campbell T.M., Hopkinson D., Kingsbury S.R., Bowes M.A., Conaghan P.G. (2015). A systematic review of the relationship between subchondral bone features, pain and structural pathology in peripheral joint osteoarthritis. Arthritis Res. Ther..

[14] MallikarjunaSwamy S., M, Holi S., M (2012). Knee joint articular cartilage segmentation, visualization and quantification using image processing techniques: a review. Int. J. Comput. Appl..

[15] Tiulpin A., Thevenot J., Rahtu E., Lehenkari P., Saarakkala S. (2018). Automatic knee osteoarthritis diagnosis from plain radiographs: a deep learning-based approach. Sci. Rep..

[16] Kellgren J.H., Lawrence J.S. (1957). Radiological assessment of osteo-arthrosis. Ann. Rheum. Dis..

[17] Kohn M.D., Sassoon A.A., Fernando N.D. (2016). Classifcations in brief: KellgrenLawrence classifcation of osteoarthritis. Clin. Orthop. Relat. Res..

[18] Schmidt J.E., Amrami K.K., Manduca A., Kaufman K.R. (2005). Semi-automated digital image analysis of joint space width in Fig. 6 Performance comparison with existing approaches 0 0.5 1 1.5 Accuracy Sensitivity Specificity Dice score 406 La radiologia medica (2022) 127:398–406 1 3 knee radiographs. Skeletal Radiol..

[19] Shamir L., Orlov N., Eckley D.M., Macura T., Johnston J., Goldberg I.G. (2008). Wndchrm-an open source utility for biological image analysis. Source Code Biol. Med..

[20] Yoo T.K., Kim D.W., Choi S.B., Oh E., Park J.S. (2016). Simple scoring system and artifcial neural network for knee osteoarthritis risk prediction: a cross-sectional study. PLoS One.

[21] Teo J.C., Mohd Khairuddin I., Mohd Razman M.A., Abdul Majeed A.P.P., Mohd Isa W.H. (2022). Automated detection of knee cartilage region in X-ray image. Mekatronika.

[22] Sikkandar M.Y., Sabarunisha Begum S., Alkathiry A.A., Alotaibi M.S.N., Manzar M.D. (2022). Automatic detection and classification of human knee osteoarthritis using convolutional neural networks. Comput. Mater. Continua (CMC).

[23] Subramoniam M., Rajini V. (2013). Local binary pattern approach to the classification of osteoarthritis in knee X-ray images. Asian Journal of Scientific Research.

[24] Mahum R., Rehman S.U., Meraj T., Rauf H.T., Irtaza A., El-Sherbeeny A.M., El-Meligy M.A. (2021 Sep 15). A novel hybrid approach based on deep CNN features to detect knee osteoarthritis. Sensors.

[25] Aladhadh S., Mahum R. (2023). Knee osteoarthritis detection using an improved CenterNet with pixel-wise voting scheme. IEEE Access.

[26] Yunus U., Amin J., Sharif M., Yasmin M., Kadry S., Krishnamoorthy S. (2022). Recognition of knee osteoarthritis (KOA) using YOLOv2 and classification based on convolutional neural network. Life.

[27] Pongsakonpruttikul N. (2022). Artificial intelligence assistance in radiographic detection and classification of knee osteoarthritis and its severity: a cross-sectional diagnostic study. Eur. Rev. Med. Pharmacol. Sci..

[28] Tiulpin A., Thevenot J., Rahtu E., Lehenkari P., Saarakkala S. (2018). Automatic knee osteoarthritis diagnosis from plain radiographs: a deep learning-based approach. Sci. Rep..

[29] Yao J., Ziapour A., Toraji R., NeJhaddadgar N. (2022). Assessing puberty-related health needs among 10-15-year-old boys: a cross-sectional study approach. Arch. Pediatr..

[30] Geng J., Ul Haq S., Ye H., Shahbaz P., Abbas A., Cai Y. (2022). Survival in pandemic times: managing energy efficiency, food diversity, and sustainable practices of nutrient intake amid COVID-19 crisis. Front. Environ. Sci..

[31] Hafeez A., Dangel W.J., Ostroff S.M., Kiani A.G., Glenn S.D., Abbas J., Mokdad A.H. (2023). The state of health in Pakistan and its provinces and territories, 1990-2019: a systematic analysis for the Global Burden of Disease Study 2019. Lancet Global Health.

[32] Bany Muhammad M., Yeasin M. (2021 Jul 12). Interpretable and parameter optimized ensemble model for knee osteoarthritis assessment using radiographs. Sci. Rep..

[33] Shamir Lior, Shari M.Ling, Scott William W., Bos Angelo, Orlov Nikita, Macura Tomasz, Mark D., Ferrucci Luigi, Ilya G. (2008).

[34] Castillo D., Cueva J., Díaz P., Lakshminarayanan V. (2023). Lecture Notes in Networks and Systems.

[35] Kellgren J.H., Lawrence J.S. (1957). Radiological assessment of osteo-arthrosis. Ann. Rheum. Dis..

[36] Kohn M.D., Sassoon A.A., Fernando N.D. (2016). Classifications in brief: kellgren-lawrence classification of osteoarthritis. Clin. Orthop. Relat. Res..

[37] Abbas J. (2021). Crisis management, transnational healthcare challenges and opportunities: the intersection of COVID-19 pandemic and global mental health. Research in Globalization.

[38] Aqeel M., Rehna T., Shuja K.H. (2022). Comparison of students' mental wellbeing, anxiety, depression, and quality of life during COVID-19's full and partial (smart) lockdowns: a follow-up study at a 5-month interval. Front. Psychiatr..

[39] Schmidt C.A., Cromwell E.A., Hill E., Donkers K.M., Schipp M.F., Johnson K.B., Hay S.I. (2022). The prevalence of onchocerciasis in Africa and Yemen, 2000-2018: a geospatial analysis. BMC Med..

[40] cgmh-oa.” Available:https://www.kaggle.com/datasets/tommyngx/cgmh-oa..

[41] Chen Pingjun (2018).

[42] Van Rikxoort E.M., Van Ginneken B. (2013). Automated segmentation of pulmonary structures in thoracic computed tomography scans: a review. Phys. Med. Biol..

[43] An J., Joe I. (2022). Attention map-guided visual explanations for deep neural networks. Appl. Sci..

[44] Selvaraju R.R., Cogswell M., Das A., Vedantam R., Parikh D., Batra D. (2016).

[45] Tiulpin A. (2019). Multimodal machine learning-based knee osteoarthritis progression prediction from plain radiographs and clinical data. Sci. Rep..

[46] Leung K. (2020). Prediction of total knee replacement and diagnosis of osteoarthritis by using deep learning on knee radiographs: data from the osteoarthritis initiative. Radiology.

[47] Bora D.J., Gupta A.K., Khan F.A. (2015).

[48] Abdullah-Al-Wadud, M.; Kabir, H.; Dewan, M.A.A.; Chae, O. A dynamic histogram equalization for image contrast enhancement. IEEE Trans. Consum. Electron..

[49] Chauhan S., Ghosh P.P.K., Shishodia M. (2010). Analysis of power amplifier by frontier recognition and histograms. Int. J. Comput. Sci. Inf. Technol..

[50] Pech-Pacheco J.L., Cristóbal G., Chamorro-Martinez J., Fernández-Valdivia J. (2000). Proceedings 15th International Conference on Pattern Recognition. ICPR-2000.

[51] Pertuz S., Puig D., Garcia M.A. (2013). Analysis of focus measure operators for shape-from-focus. Pattern Recogn..

[52] Prezja F., Paloneva J., Pölönen I., Niinimäki E., Äyrämö S. (2022). DeepFake knee osteoarthritis X-rays from generative adversarial neural networks deceive medical experts and offer augmentation potential to automatic classification. Sci. Rep..

[53] Azam S., Rafid A.R.H., Montaha S., Karim A., Jonkman M., De Boer F. (2023). Automated detection of broncho-arterial pairs using CT scans employing different approaches to classify lung diseases. Biomedicines.

[54] Pratik Kalshetti, Manas Bundele, Parag Rahangdale, Dinesh Jangra, Chiranjoy Chattopadhyay, Gaurav Harit and Abhay Elhence, An Interactive Medical Image Segmentation Framework Using Iterative Refinement,Comput. Biol. Med., 10.1016/j.compbiomed.2017.02.002. 28214717

[55] Types of morphological operations - MATLAB & Simulink,”*Mathworks.com*. [Online]. Available: https://www.mathworks.com/help/images/morphological-dilation-and-erosion.html. [Accessed: 11-May-2023]..

[56] Lingayat N.S., Tarambale M.R. (2013). A computer based feature extraction of lung nodule in chest X-ray image. Int. J. Biosci. Biochem. Bioinforma..

[57] Mohamed Hend, Omar Rowan, Saeed Nermeen, Essam Ali, Ayman Nada, Mohiy Taraggy, AbdelRaouf Ashraf (2018).

[58] Rafid A.K.M.R.H., Azam S., Montaha S., Karim A., Fahim K.U., Hasan M.Z. (2022). An effective ensemble machine learning approach to classify breast cancer based on feature selection and lesion segmentation using preprocessed mammograms. Biology.

[59] Iqbal N., Mumtaz R., Shafi U., Zaidi S.M.H. (2021). Gray level co-occurrence matrix (GLCM) texture based crop classification using low altitude remote sensing platforms. PeerJ Comput. Sci..

[60] Yan X., Gao L. (2020). A feature extraction and classification algorithm based on improved sparse auto-encoder for round steel surface defects. Math. Biosci. Eng..

[61] Sun Shiyu, Xue Wufeng, Zhou Yongjin (2020). Classification of young healthy individuals with different exercise levels based on multiple musculoskeletal ultrasound images. Biomed. Signal Process Control.

[62] Ghosh P. (2021). Efficient prediction of cardiovascular disease using machine learning algorithms with relief and LASSO feature selection techniques. IEEE Access.

[63] Fatema K., Montaha S., Rony M.A.H., Azam S., Hasan M.Z., Jonkman M. (2022). A robust framework combining image processing and deep learning hybrid model to classify cardiovascular diseases using a limited number of paper-based complex ECG images. Biomedicines.

[64] Calhoun V.D., Adali T. (2009). Feature-based fusion of medical imaging data. IEEE Trans. Inf. Technol. Biomed..

[65] Gomez-Chova L. (2004). IGARSS 2003. 2003 IEEE International Geoscience and Remote Sensing Symposium. Proceedings.

[66] Parveen A.N., Inbarani H.H., Kumar E.N.S. (2012). 2012 International Conference on Computing.

[67] Biswas S., Bordoloi M., Purkayastha B. (2016). Review on feature selection and classification using neuro-fuzzy approaches. Int. J. Appl. Evol. Comput..

[68] Abbas J. (2020). The impact of coronavirus (SARS-CoV2) epidemic on individuals mental health: the protective measures of Pakistan in managing and sustaining transmissible disease. Psychiatr. Danub..

[69] Micah A.E., Bhangdia K., Cogswell I.E., Lasher D., Lidral-Porter B., Maddison E.R., Dieleman J.L. (2023). Global investments in pandemic preparedness and COVID-19: development assistance and domestic spending on health between 1990 and 2026. Lancet Global Health.

[70] NeJhaddadgar N., Ziapour A., Zakkipour G., Abolfathi M., Shabani M. (2020, Nov 13). Effectiveness of telephone-based screening and triage during COVID-19 outbreak in the promoted primary healthcare system: a case study in Ardabil province, Iran. Z Gesundh Wiss.

